# Physical and Electrochemical Analysis of *N*-Alkylpyrrolidinium-Substituted Boronium Ionic Liquids

**DOI:** 10.1021/acs.inorgchem.3c02971

**Published:** 2023-10-23

**Authors:** Christopher D. Stachurski, James H. Davis, Tyler Cosby, Margaret E. Crowley, Nathaniel E. Larm, Mollie G. Ballentine, Richard A. O’Brien, Matthias Zeller, E. Alan Salter, Andrzej Wierzbicki, Paul C. Trulove, David P. Durkin

**Affiliations:** † Department of Chemistry, U.S. Naval Academy, Annapolis, Maryland21402, United States; ‡ Department of Chemistry, University of South Alabama, Mobile, Alabama36688, United States; § School of Mathematics and Sciences, University of Tennessee Southern, Pulaski, Tennessee38478, United States; ∥ Department of Chemistry, Purdue University, West Lafayette, Indiana47907, United States

## Abstract

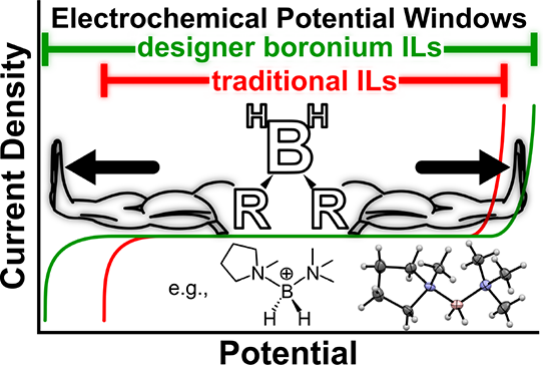

In this work, a series of novel boronium-bis(trifluoromethylsulfonyl)imide [TFSI^–^] ionic liquids (IL) are introduced and investigated. The boronium cations were designed with specific structural motifs that delivered improved electrochemical and physical properties, as evaluated through cyclic voltammetry, broadband dielectric spectroscopy, densitometry, thermogravimetric analysis, and differential scanning calorimetry. Boronium cations, which were appended with *N*-alkylpyrrolidinium substituents, exhibited superior physicochemical properties, including high conductivity, low viscosity, and electrochemical windows surpassing 6 V. Remarkably, the boronium ionic liquid functionalized with both an ethyl-substituted pyrrolidinium and trimethylamine, [(1-e-pyrr)N_111_BH_2_][TFSI], exhibited a 6.3 V window, surpassing previously published boronium-, pyrrolidinium-, and imidazolium-based IL electrolytes. Favorable physical properties and straightforward tunability make boronium ionic liquids promising candidates to replace conventional organic electrolytes for electrochemical applications requiring high voltages.

## Abstract 2

In this work, ionic liquids centered on novel “boronium” cations are prepared and characterized utilizing a streamlined one-pot synthesis. The boronium ionic liquids reported were stable beyond 200 °C and exhibited remarkable electrochemical stability, many of which surpassing 6 V. These compounds represent a monumental push forward toward designer IL electrolytes whose properties can be tuned through careful selection of ligand donors during synthesis.

## Introduction

Since their inception, ionic liquids (ILs) have proven to be increasingly versatile as solvents and electrolytes with each new class of material discovered.^1,2^ When considering ILs for electrochemical applications, their inherent ionic conductivity, low vapor pressure and flammability, and relatively high thermal stability at electrochemically relevant temperatures make them competitive alternatives to organic electrolyte systems.^1^ The majority of ILs studied for these applications to date include cations consisting of aromatic and saturated cyclic amines (e.g., imidazolium and pyrrolidinium), sulfoniums, and quaternary phosphoniums paired with inorganic (chloroaluminates) or organic (e.g., triflate, bistriflimide, and acetate) anions.^3−5^ One of the more prominent classes of IL electrolytes reported in the literature combines *N*,*N*-dialkylpyrrolidinium cations with bisfluorosulfonylimide (FSI^–^) or bis(trifluoromethansulfonyl)imide (TFSI^–^) anions.^6^ These ILs have exceptionally high electrochemical windows (ca. 5.5–6.0 V), surpassing what can be achieved in a conventional organic electrolyte.^7^ For example, *N*-butyl-*N*-methylpyrrolidinium TFSI ([14pyrr][TFSI]) exhibits high ionic conductivity and cathodic stability, supporting its potential as an electrolyte for battery and supercapacitor applications.^8,9^ While great progress has been made with the existing body of ILs, further exploration is necessary to identify new systems with higher voltage windows and stability (chemical, electrochemical, and thermal) to fully address the growing needs of energy storage.

**Scheme 1:**
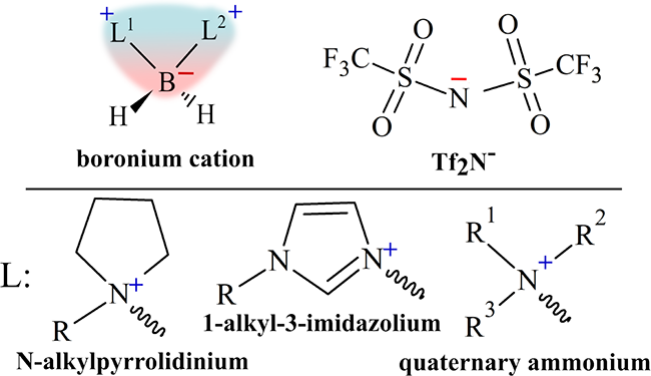
General Structure of boronium Ionic liquid Highlighting the Zwitterionic Charge Distribution across the Cation Backbone

An understudied class of ILs are those incorporating boronium cations, often in the form [L^1^L^2^-BH_2_]^+^, where L^1^ and L^2^ can be one of many different Lewis basic organic moieties (Scheme 1). These boronium ionic liquids (BILs) were first introduced in 2005 by Davis et al., who highlighted the synergetic combination of hydrophobicity, low melting point, and structural versatility afforded by the [L^1^L^2^BH_2_]^+^ formula, which imparts a zwitterionic charge distribution across the cation backbone.^10^

Shortly after their introduction, Rüther et al. evaluated the potential for using BILs as electrolyte candidates in lithium ion batteries.^11^ BILs with the cations [(1-methylimidazolium)_2_BH_2_]^+^, [(1-butylimidazolium)_2_BH_2_]^+^, and [N_111_N_112_BH_2_]^+^ (N_111_ = trimethylamine; N_112_ = dimethylethylamine) and the [TFSI]^−^ anion were compared to *N*-butyl-*N*-methylpyrrolidinium TFSI ([14pyrr][TFSI]), an established IL electrolyte. In particular, the bis(amine) BIL exhibited a slightly superior electrochemical window (5.8 V) over the dialkylpyrrolidinium analogue (5.5 V) while sustaining reversible lithium electrodeposition, serving as an initial proof of concept for using BILs as electrolytes. Despite their superior electrochemical stability, there have only been a few detailed BILs studies in the following years.

Rüther et al. conducted a focused investigation on the impact of alternate anions like bis(fluorosulfonyl)imide (FSI^–^) and the presence of lithium salt on the physicochemical properties of bis(amine) boronium cation-containing ILs.^11^ In addition, McCallum et al. reported that the longevity of Li/Li cells containing BIL electrolytes was attributed to the different reactivities of BIL from conventional pyrrolidinium IL systems at the electrode surface.^12^ Both ILs have been shown to induce drastically different anion coordination environments for Li^+^, with BILs offering a more stable arrangement of species in the resulting solid-electrolyte interface (SEI) layer. This theory was later verified by Clarke-Hannaford et al. through density functional theory (DFT) calculations, which demonstrated how anionic species could readily chemisorb and degrade at Li(001) surfaces creating a stable SEI layer, which is crucial to sustained cycling of Li-containing electrochemical cells.^13^ Clarke-Hannaford et al. extended this work to the (*N*,*N*,*N’*,*N’*-tetramethylethylenediamine)dihydroborate cation, a cyclic derivative of the bis(amine) structure, again revealing cationic stability toward degradation at Li(001) surfaces despite significant modification of the cation structure.^14^ In addition to serving as electrolytes, BILs have also been identified as hypergolic fuels boasting high energy densities, low ignition delays, and low viscosities. Still further, select boronium iodide salts have shown significant antibacterial, antifungal, and antiviral activity, all of these factors attesting to the diverse potential of this novel class of cations.^15−19^

These foundational studies demonstrate that BILs can possess enhanced electrochemical stability when compared to common IL electrolytes—likely due to the unique electronic distribution across the boronium cation—which facilitates tailoring of the pendant cationic L^1^ and L^2^ moieties for favorable physical, thermal, and electrical properties. To test this versatility, several novel boronium cations incorporating trialkylamine and *N*-alkylpyrrolidinium moieties were prepared and characterized using a series of physical, thermal, and electrochemical methods.

## Experimental Section

### Materials

**Figure 1:**
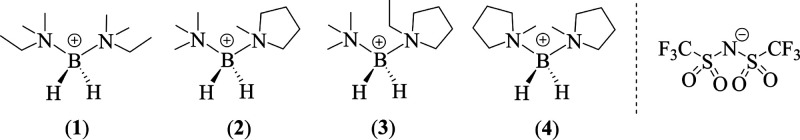
Structure of the characterized boronium cations [N_112_N_112_BH_2_] (**1**), [(1-m-pyrr)N_111_BH_2_] (**2**), [(1-e-pyrr)N_111_BH_2_] (**3**), and [(1-m-pyrr)_2_BH_2_] (**4**). Following synthesis, the iodide salts of each BIL were anion-exchanged with [TFSI]^−^.

The boronium ionic liquids (BILs **1**–**4**; Figure 1) were synthesized and characterized, as described in the Supporting Information. (Caution! Many of the additions described in the synthetic procedure are highly exothermic and lead to the vigorous evolution of H_2_. All steps should be performed slowly to control the evolution of heat and H_2_, as denoted in the Supporting Information). The original IL mixtures were first synthesized as iodide salts and then underwent anion exchange with TFSI. Prepared ILs were dried at 60 °C under vacuum prior to use to remove any residual solvent. The IL 1-ethyl-3-methyl imidazolium bis(trifluoromethane)sulfonimide ([EMI][TFSI]; Iolitec, IL-0023-HP-0100) was stored under a dry inert atmosphere and used as received. Lithium trifluoromethanesulfonimide (Li[TFSI]; 3M, lot no. 10092) was dried at 60 °C under vacuum before transferring to a glovebox for storage and dissolution in select BIL electrolytes. Silver triflate (Sigma-Aldrich, 2923-28-6) was stored under nitrogen and kept in the dark when not in use to minimize decomposition.

### Characterization

A brief summary of the methods used to characterize the produced BILs is provided in the manuscript. Complete experimental details can be found in the Supporting Information.

#### Thermal Characterization

Thermogravimetric analysis (TGA) was used to determine the thermal stability of each BIL under nitrogen. Measurements were made on a TA Instruments Q500 instrument with constant heating from 20 to 900 °C.

Differential scanning calorimetry (DSC) was used to identify relevant thermal phase transitions of each BIL using a TA Instruments Q2000. Samples were cycled twice over the reported temperature range, and the phase transitions were measured from the heating curves of each sample.

#### Single-Crystal Structure Determinations

Crystal structures of the BPh_4_ salts of BIL **2** and **4** (Figure 1) were determined by single-crystal X-ray diffraction using a Bruker Quest diffractometer with a fixed chi angle, a Mo Kα wavelength (λ = 0.71073 Å) sealed tube fine focus X-ray tube, single-crystal curved graphite incident beam monochromator, a Photon II area detector, and an Oxford Cryosystems low-temperature device. CCDC 2292365 and 2292366 contain the supplementary crystallographic data for this paper. These data can be obtained free of charge from the Cambridge Crystallographic Data Centre via www.ccdc.cam.ac.uk/data_request/cif.

#### Electrochemical Characterization

Electrochemical measurements, including the electrochemical solvent window and the cycling of Li^+^, were performed within a nitrogen-filled glovebox using a Biologic SP-200 potentiostat. All samples were analyzed using a three-electrode setup consisting of either a glassy carbon or a platinum working electrode (EDAQ, surface area = 7.8 × 10^–3^ cm^2^), a platinum mesh counter electrode, and a home-built Ag/Ag^+^ reference electrode made from a 100 mM solution of silver triflate dissolved in [EMI][TFSI] contained within a glass tube sealed with a vycor glass frit.

Electrostatic potential energy maps of BIL cations **2**–**4** (Figure 1) and other cations of interest, along with their reduced daughter species (charge = 0, multiplicity = 2), were optimized using the Gaussian16 suite of programs^20^ with the wB97X-D density functional^21^ and the cc-pvtz basis set.^22^

Broadband dielectric spectroscopy (BDS) was used to study the temperature-dependent dielectric response across a range of frequencies. Measurements were made using a Novocontrol α-analyzer and HP E4991B impedance analyzer with a Novocontrol RF extension for low and high frequency ranges, respectively.

#### Density and Viscosity Characterization

Temperature-dependent densities and kinematic-dynamic viscosities were measured using an SVM 3001 Stabinger viscometer (Anton Paar) with temperature controlling capabilities.

The temperature-dependent zero-shear viscosities were measured using a Discovery HR-2 rheometer (TA Instruments), operated under nitrogen with 25 mm stainless steel parallel plates and stress/rate-controlled flow experiments.

## Results and Discussion

### Synthesis of *N*-Alkylpyrrolidinium Boronium Cation-Based ILs

**Scheme 2:**
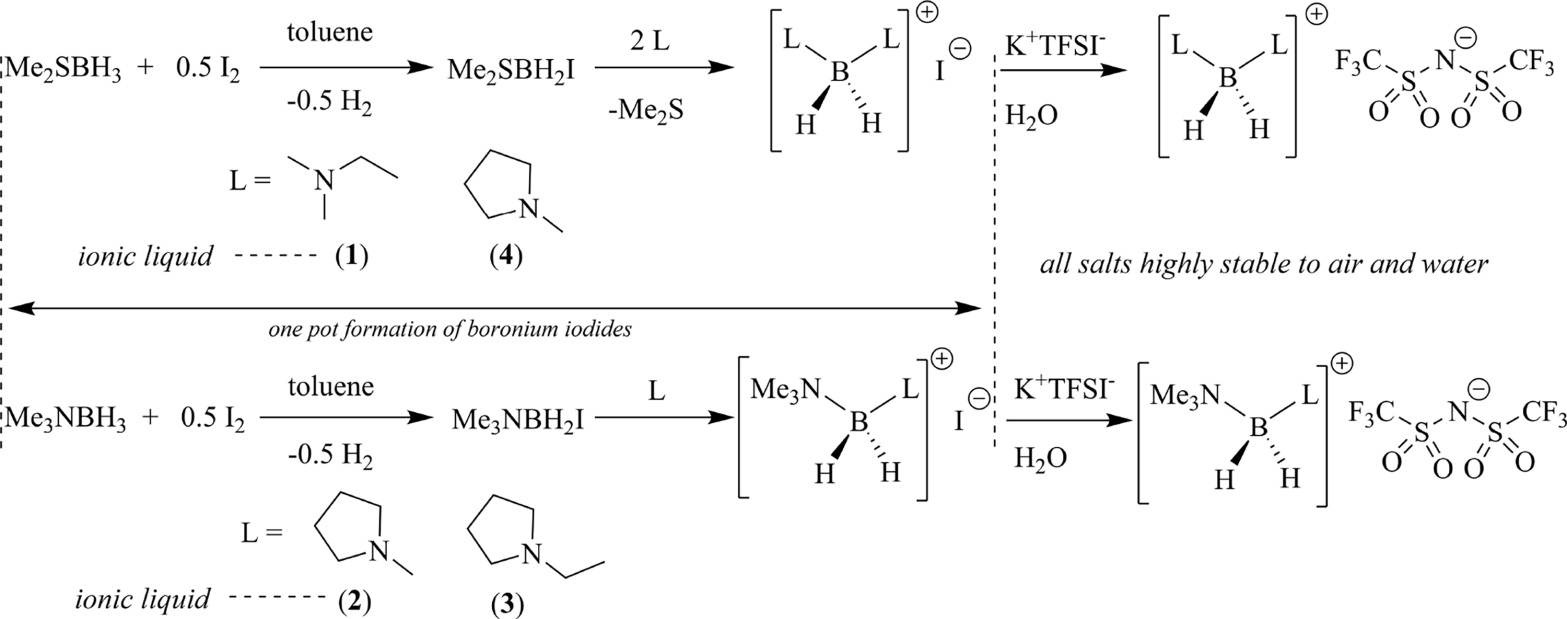
Synthetic Scheme for BILs **1**–**4**

The boronium ions in ionic liquids **1**–**4** were initially prepared as their iodide salts using one of two different but related approaches (Scheme 2), each of which is carried out in air. In salts **2** and **3**, the boron center of the cation is ligated by two different amines, one of which in each salt is trimethylamine.

These were prepared using a protocol introduced by Douglass^23^ and refined by Ryschkewitch et al.,^24^ beginning with easily handled, commercially available Me_3_NBH_3_ (trimethylamine borane). When the trimethylamine borane complex is dissolved in toluene and treated with I_2_, it undergoes spontaneous evolution of H_2_ with the concomitant *in situ* formation of Me_3_NBH_2_I. Once the reaction is complete (ca. 30 min, evidenced by the return of the solution to a colorless or near-colorless state), 1 equiv of the desired second amine (*N*-methylpyrrolidine in the case of **2** and *N*-ethylpyrrolidine in the case of **3**) is added in one portion, bringing about the formation of the desired products as white precipitates, which are isolated by filtration and dried in vacuo. Subsequent dissolution of the iodide salts in water, followed by the addition of K[TFSI] [potassium bis(trifluoromethane sulfonylimide)], induces in each case the formation of a dense, colorless layer of the product liquid, which is separated from the aqueous phase and dried in vacuo to remove any remaining water.

The synthesis of BILs **1** and **4**, each of which incorporates two molecules of a single type of amine, follows a similar course but beginning from Me_2_SBH_3_ (dimethylsulfide borane) to better facilitate the disubstitution. Here again, a toluene solution of the borane complex is treated with I_2_ to form an iodoborane intermediate, Me_2_SBH_2_I [note: unlike in the aforementioned reactions, the intermediate solution color rarely returns to colorless, tending to remain somewhat orange even after addition of the amine to it]. To this intermediate are then added 2 equiv of the desired amine (ethyldimethylamine in the case of **1** and *N*-methylpyrrolidine in the case of **4**). These react with the intermediate over time by displacing from the boron center both I^–^ and dimethylsulfide. In these cases, Me_3_NBH_2_I is not used as the synthetic intermediate because, unlike Me_2_S, the replacement of the B-bonded Me_3_N ligand is slow, requiring forcing conditions.

**Figure 2:**
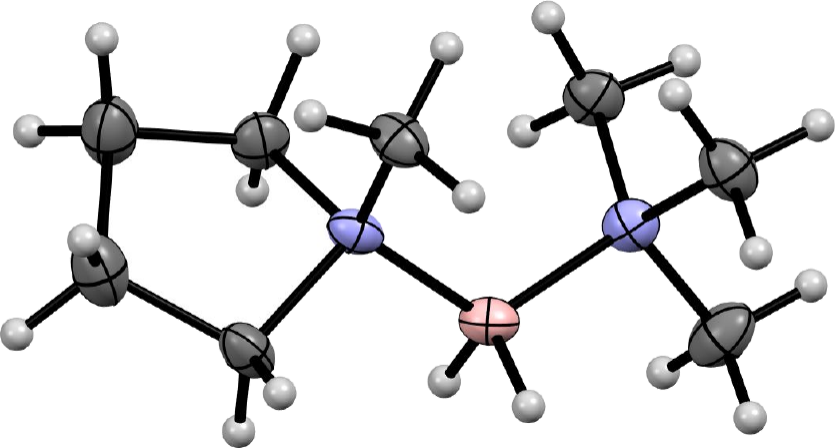
Displacement ellipsoid plot (displacement ellipsoid probabilities of 50% for non-hydrogen atoms) of [(1-m-pyrr)N_111_BH_2_], the cation of BIL **2**, clearly showing the coordination to the boron center of the supporting *N*-methylpyrrolidine and trimethylamine ligands. A second conformer, also present and disordered with that shown, is omitted for clarity, as is the BPh_4_^–^ anion used to obtain a crystalline salt with this cation.

In addition to the preparation of the I^–^ and [TFSI]^−^ salts of cations **1**–**4**, BPh_4_^–^ salts of **2** (Figure 2) and **4** (Figure S1) were also prepared. Upon dissolution in hot methanol/acetone and cooling, suitable single crystals were obtained and studied by single-crystal X-ray diffraction, providing further validation of the proposed boronium cation structures (*vide supra*).

### Thermal Characterization of *N*-Alkylpyrrolidinium Boronium Cation-Based ILs

Thermal properties of the prepared boronium ILs were studied by using differential scanning calorimetry (DSC) and thermogravimetric analysis (TGA). The relevant thermal values for each material are provided in Table 1.

**Table 1:** Thermal and Electrochemical Properties of BILs **1**–**4**^a^

BIL	*T*_g_ (°C)	*T*_c_ (°C)	*T*_m_ (°C)	*T*_5_ (°C)	σ (mS cm^–1^)	*E*_ox_ (V)	*E*_red_ (V)	Δ*E* (V)
(**1**) [N_112_N_112_BH_2_]	–90.7	–31.6	5.6	202	1.8	+2.1	–3.9	6.0
(**2**) [(1-m-pyrr)N_111_BH_2_]	–72.3	–35.0	18.6	251	1.8	+2.2	–3.9	6.1
(**3**) [(1-e-pyrr)N_111_BH_2_]	–72.9	–11.2	–0.1	217	1.3	+2.1	–4.2	6.3
(**4**) [(1-m-pyrr)_2_BH_2_]	–68.1	–28.1	10.3	259	1.5	+2.1	–4.0	6.1

a *T*_g_, *T*_c_, and *T*_m_ were measured from DSC heating curves from −150 to 50 °C at 10 °C min^–1^; *T*_5_ was measured using TGA and determining the point at which 5 wt % of the initial sample mass was lost; σ was determined from BDS measurements at 300 K; *E*_ox_ and *E*_red_ were measured using CV; Δ*E* is reported as the difference between *E*_ox_ and *E*_red_.

**Figure 3:**
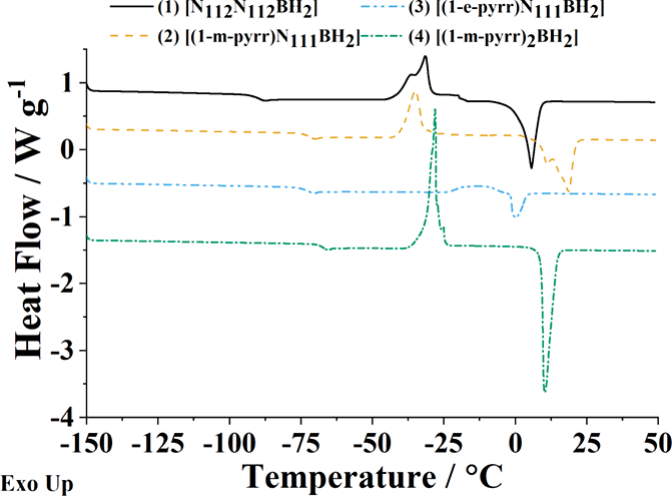
Differential scanning calorimetry heating traces of BILs **1**–**4**. Samples were first preconditioned at 50 °C and then cooled to −150 °C before heating to 50 °C at 10 °C min^–1^. The onset glass transition temperatures (*T*_g_) were determined for each sample and are provided in Table 1.

Despite structural differences across the suite of BILs tested, heating/cooling ramps over the temperature range of −150 to 50 °C for each sample show similar thermal behavior regardless of the cation structure (Figure 3).

Each BIL undergoes a single glass transition at low temperatures (less than −60 °C) consistent with many previously studied IL systems, including past BILs.^3,11^ Of the BILs prepared, **1** exhibits a significantly lower *T*_g_ (−91.07 °C) than those containing *N*-alkylpyrrolidinium moieties. The *T*_g_ of BIL **1** (containing two dimethylethylamine ligands) is slightly lower than that of the previously studied analogue [(N_111_)(N_112_)BH_2_][TFSI] (*T*_g_ reported as −79.2^11^ and −83.0 °C^12^, respectively). Many factors are at play when considering the glass-forming behavior of ILs, including charge distribution across the ions as well as cation–anion interactions. Generally, the *T*_g_ of these boronium ILs tend to increase with ion size, length of alkyl groups, and symmetry of the constituent ions, similar to what has been observed in studies of imidazolium cation-containing ILs.^25^

While the addition of a second dimethylethylamine to the cation increases both the size and symmetry of the IL, the observed decrease in *T*_g_ could be the result of increased crowding at the second cationic amine group, leading to hindered interactions between the [TFSI]^−^ anion and the cationic regions of BIL **1**. The increased number of rotational degrees of freedom provided by the second ethyl group (versus a methyl group) may also contribute to this effect, facilitating the conformational reorganization needed to invoke such phase transitions.^26^ BILs **2**–**4** exhibit similar *T*_g_s (**2** = −72.3 °C, **3** = −72.9 °C, and **4** = −68.1 °C), all of which are lower than their dialkylpyrrolidinium cation-containing TFSI analogues.^11,12^ From the samples tested, no substantial change in *T*_g_ was observed when the alkyl chain length was extended from one to two carbons or the trialkylamine was replaced with a second *N*-alkylpyrrolidinium moiety. BILs **1**–**4** also demonstrate the capacity to undergo crystallization (*T*_c_) and melting (*T*_m_) events following glass transitions.^27^ BIL **1** exhibits a large temperature gap between crystallization and melting (*T*_c_ = −31.6 °C, *T*_m_ = 5.6 °C), consistent with previously studied analogues.^11^ The remaining BILs exhibit similar thermal behavior from a supercooled state, as seen with BIL **1**. Notably, when two *N*-alkylpyrrolidinium moieties were conjugated to the boronium cation, an increase in *T*_c_ (−35.0 to −28.1 °C) and decrease in *T*_m_ (18.6 to 10.3 °C) were observed, suggesting overall decreased stability of the crystalline intermediate for BIL **4**. A similar, more pronounced effect was observed following elongation of the alkyl group on the pyrrolidinium moiety from a methyl to an ethyl, as seen between BILs **2** and **3**, with both phase changes shifting nearly 20 °C closer in value (*T*_c_ = −11.2 °C, *T*_m_ = −0.1 °C). Past research on alternative alkyl-substituted IL systems (i.e., imidazolium and pyrrolidinium) has highlighted the significance of chain length on phase behavior, which is also to be expected for this suite of BILs.^27^

It is worth noting that repeat cycling of the BILs reveals inconsistencies in the crystallization and melting behavior (Figure S2). Similar behavior has been seen with other IL systems in the literature, including BILs, and has been attributed to fragility of the crystalline activity due to metastable solid phases of the material in the measured temperature ranges.^12^ This behavior should be considered when evaluating these materials for future applications as standalone solid-state electrolytes.^11,12,28,29^ Importantly, all BILs tested in this study demonstrate remarkable stability at room temperature without any evidence of crystallization following prolonged storage in the glovebox.

When BILs are considered for temperature-dependent energy storage applications, the thermal stability must be understood. BILs **1**–**4** exhibit high thermal stability, withstanding temperatures up to at least 200 °C before experiencing significant loss of mass during heating under nitrogen (Figure S3). By substituting *N*-methylpyrrolidiniums for the trialkylamine pendent group on the boron center, *T*_5_ increased by 49 and 57 °C for one (BIL **2**; *T*_5_ = 251 °C) and two (BIL **4**; *T*_5_ = 259 °C) substitutions, respectively. This trend agrees with the hypothesized mechanism of thermal decomposition of BILs, in which amine-based donors can be easily displaced from the boron center by anionic species in the salt leading to their comparatively lower *T*_5_.^11,30^ A significant decrease in *T*_5_ from 251 to 217 °C was observed but between BILs **2** and **3** as the length of the alkyl chain increased. Similar behavior was observed for analogous dialkylimidazolium-based ILs, with the onset decomposition temperature (*T*_onset_) decreasing at least 10 °C upon increasing the alkyl chain length from two to four carbons and in *N*-alkyl-*N*-methyl pyrrolidinium ILs [1*n*pyrr][TFSI] ILs, where *T*_onset_ decreased by up to 48 °C as *n* increased from 3 to 10 carbon atoms.^31−33^ Overall, the BILs presented in this study show thermal stability above 200 °C, comparable to many IL electrolytes previously applied toward electrochemical energy storage.^34^

### Electrochemical Analysis of *N*-Alkylpyrrolidinium Boronium Cation-Based ILs

**Figure 4:**
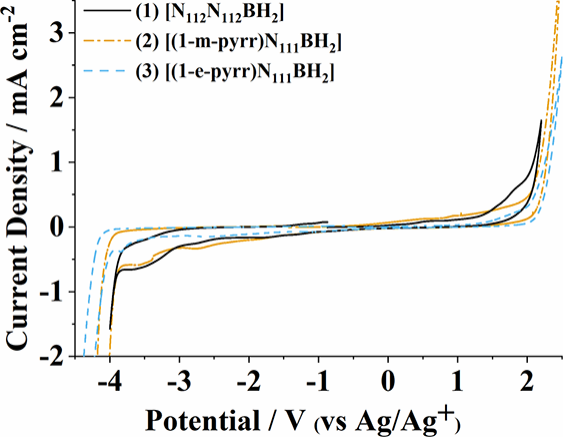
Representative cyclic voltammograms of BILs **1**–**3**. All scans were conducted at 50 mV s^–1^ from *V*_ocp_, which consistently hovered around −0.8 to −1 V, to adequate switching potentials determined through iterative preliminary CVs. In the voltammograms shown, BILs **1** and **2** were swept p–n while BIL **3** is shown under n–p scanning. All scans were collected at a glassy carbon working electrode polished between scans.

Prior to applying new IL materials toward electrochemical applications, it is important to identify the range of stable working potentials accessible with each new ion pair.^11,35−37^ The absolute electrochemical windows of BILs **1**–**4** were measured with a glassy carbon electrode (GCE) at room temperature, which was cycled from the open circuit potential (*E*_ocp_) to successively greater positive and negative potentials until a benchmark current density of 1 mA cm^–2^ was surpassed (Figure 4 and Table 1 and Figure S4).

Previous studies investigating the electrochemical stability of BILs have utilized a range of working electrode materials (i.e., platinum, gold, and boron-doped diamond) at different scan rates, all of which will impact the onset potentials for decomposition of the anion and cation and should be considered when comparing results between studies.^11,12^ GCEs were selected for initial electrochemical solvent window screening for their high overpotential toward hydrogen reduction and similarity to carbon materials used in electrochemical energy storage.

Each BIL tested in this study demonstrates oxidative stability up to roughly +2 V (vs Ag/Ag^+^) (Table 1). Since the [TFSI]^−^ anion was used across samples, the limiting oxidation potential was expected to be consistent between each electrolyte. The slight variations in *E*_ox_ observed across BILs **1**–**4** are attributed to differences in the cation-TFSI interaction, with some cationic structures leading to more stable molecular arrangements of the ion pair that may protect either species from oxidative decomposition.^1^ When sweeping toward negative working potentials, BIL **1** exhibits an electrochemical stability to −3.9 V (vs Ag/Ag^+^), comparable to a previously published analogue [N_111_N_112_BH_2_][TFSI], which was stable to −3.8 V (vs Ag/Ag^+^) at a platinum working electrode.^11^ BILs containing *N*-alkylpyrrolidinium moieties (BILs **2** and **3**) demonstrated greater electrochemical stability, notably reaching cathodic potentials beyond −4.0 V (vs Ag/Ag^+^). This trend was not followed upon the addition of a second *N*-alkylpyrrolidinium moiety (BIL **3** vs BIL **4**), highlighting how the second cationic moiety could be selected to influence the bulk properties of the electrolyte (Figure S4).

To date *N*,*N*-dialkylpyrrolidiniums (i.e., [14pyrr][TFSI]) have been the primary class of ILs capable of exhibiting electrochemical stabilities comparable to the BIL electrolytes presented in this study (c.a. 5.6 V).^12,37^ Yet, pyrrolidinium ILs experience limitations in their synthetic derivatization, restricted to altering the two alkyl side chains, compared to the [L^1^L^2^BH_2_] template. When factoring in the limiting oxidation potential for each respective BIL, electrochemical solvent windows for BILs **2** and **3** reach an impressive 6.1 and 6.3 V, respectively (Figure 4). Studies on the electrochemical stability of dialkylimidazolium ILs by Kazemiabnavi et al. have shown that increasing the length of the alkyl chain has minimal impact on the limiting cathodic potential as the lowest unoccupied orbital is controlled by the imidazolium ring.^4^ Appetecchi et al. conducted a similar study on *N*-alkyl-*N*-methylpyrrolidinium ILs, which found a slight extension of the limiting cathodic potential as the chain length increased (propyl: −3.7 V; heptyl: −3.9 V).^37^ Interestingly, high variability in the cathodic potential was observed across different isomers of the butyl-substitution (*n*: −3.8 V; *iso*: −4.2 V; *sec*: −1.5 V), suggesting that the alkyl chain of pyrrolidinium-modified BILs can induce significant changes in the electrochemical solvent window of the electrolyte beyond ethyl substitution, warranting further investigation.

To further understand the impact of the cation structure on electrochemical behavior, computational gas-phase reduction potentials (*E*^0^_red_) are presented in Table 2 for several BIL cations, including two analogues of the novel BIL **2** cation.

**Table 2:** Theoretical Reduction Potentials of the BIL Cations^a^

cation	*E*^0^_red_(V)
N_111_N_111_BH_2_	1.7
(**2**) [(1-m-pyrr)N_111_BH_2_]	1.6
(**3**) [(1-e-pyrr)N_111_BH_2_]	1.5
(1-m-pyrr)C_111_CH_2_	1.7
(1-m-cyclopentyl)N_111_CH_2_	1.9
(**4**) [(1-m-pyrr)_2_BH_2_]	1.5
(1-e-pyrr)_2_BH_2_	1.4

a Geometries were optimized using the wB97X-D/cc-pvtz computational model; reduction potentials are from gas-phase estimates of the Δ*H*^0^ for the reduction at 298 K.

**Figure 5:**
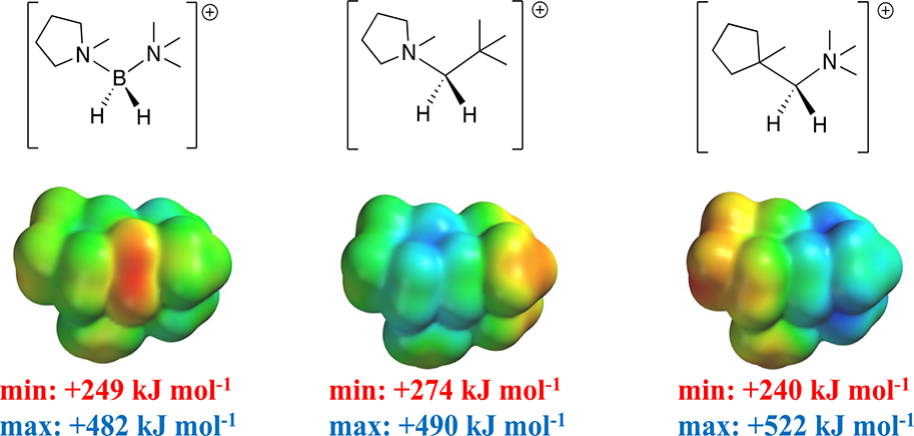
Electrostatic potential energy maps of the BIL **2** cation (left) and its alkylpyrrolidinium and alkylamine analogues. Common color range: +249 (red) to +522 (blue) kJ mol^–1^. Note how the regions of the highest and lowest electrostatic potential “move” in the three isosteric and isoelectronic ions.

The cations [(1-m-pyrr)C_111_CH_2_] and [(1-m-cycloptentyl)N_111_CH_2_] were chosen as a first step to rationalize the especially wide electrochemical window seen with BIL **2**. Higher reduction potentials observed for the pyrrolidinium [(1-m-pyrr)C_111_CH_2_] and ammonium [(1-m-cyclopentyl)N_111_CH_2_] analogues compared to BIL **2** (1.7 and 1.9 V versus 1.6 V) are consistent with the general experimental observation that windows for boronium-based *N*-alkylpyrrolidinium ILs are wider than their structural analogues. The electrostatic potential energy maps (elstats) for the series (Figure 5) illustrate greater delocalization of charge over the BIL **2** cation, largely due to the zwitterionic N–B–N moiety when compared to the modeled ammonium ion isosteres.

The maximum elstat value of BIL **2** (+482 kJ mol^–1^) is the lowest of the three, consistent with its lowest reduction potential. The same maps demonstrate how the distribution of the charge density can be deliberately varied in structurally similar species. This, in turn, likely impacts relative cation–anion interactions in close contact pairs, thus constituting another way in which the properties of these ILs can be tuned.^38^

The simple N–B–N cation [N_111_N_112_BH_2_], which was examined in depth in an earlier work,^11^ was examined next to assess the impact of the pyrrolidinium rings in BILs **2** and **3**. The methyl-substituted pyrrolidinium ring lowers the reduction potential from 1.7 V (N_111_N_111_BH_2_) to 1.6 V (BIL **2** cation), while the addition of an ethyl-substituted pyrrolidinium further decreases *E*^0^_red_ to 1.5 V (BIL **3** cation), as shown in Table 2. According to computed spin charges, the reduced form of the BIL **2** cation has greater delocalization of alpha spin density versus the reduced form of the BIL **3** cation, consistent with the greater reduction potential of BIL **2**. Interestingly, the presence of two 1-methyl pyrrolidinium rings (BIL **4** cation) yields only a modest lowering of the reduction potential over the presence of just one (BIL **2** cation), as was also observed experimentally (Table 1). To test this, we computed the reduction potential of the hypothetical [(1-e-pyrr)_2_BH_2_] cation with two 1-ethyl pyrrolidinium rings. The resulting theoretical *E*^0^_red_ value of 1.4 V is lower than that of the BIL **3** cation by 0.2 V. Natural bond order analysis indicates that the reduced form of the BIL **4** cation may be uniquely stabilized by delocalization of the spin charge across the N–B–N moiety from L_1_ to L_2_. Consequently, the reduction potential of BIL **4** is not as low as might be otherwise expected.

Short chain *N*-alkylpyrrolidinium moieties on boronium cations extend the limiting cathodic potential of the resulting electrolyte over trialkylamine and imidazolium-modified boronium cations, in addition to other common room temperature ionic liquid cations such as dialkylimidazoliums (c.a. 4.2 V),^4^ phosphoniums (c.a. 5 V),^39^ and pyrrolidiniums (c.a. 5.6 V),^40^ making them ideal for high voltage electrochemical energy storage. Furthermore, appending a secondary cationic moiety in addition to the favorable *N*-alkylpyrrolidinium could leverage the designer nature of BIL electrolytes to create electrolytes with tunable, superior properties to more conventional ILs.

### Lithium Electrochemistry of *N*-Alkylpyrrolidinium Boronium Cation-Based ILs

**Figure 6:**
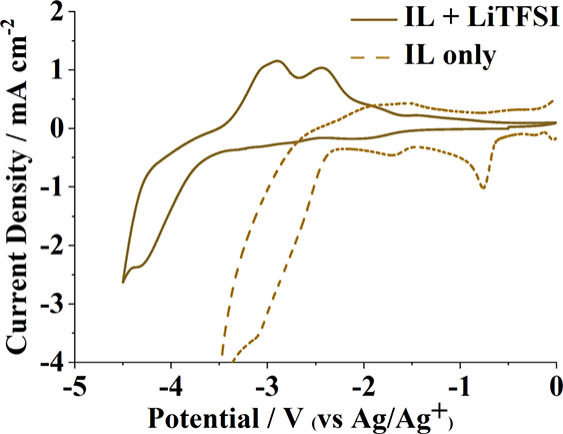
Cyclic voltammograms of BIL **2** in the presence (solid) and absence (dotted) of 0.45 mol kg^–1^ Li[TFSI]. For both scans, a platinum disk and mesh electrode were used for the working and counter electrode, respectively. The cell was held at *E*_ocp_ for 15 s before scanning at 0.05 V s^–1^, first negative and then positive before ending at *E*_ocp_.

As a preliminary test for the applicability of the synthesized BILs toward electrochemical energy storage applications, the top-performing BILs (**2** and **3**) were screened for reversible lithium plating and stripping. Cyclic voltammograms of BIL **2** solutions in the presence and absence of 0.45 mol kg^–1^ Li[TFSI] at a platinum working electrode demonstrate the stark impact additives can induce on these electrolytes (Figure 6).

In addition to suppressing the secondary oxidation and reduction signals seen in the neat IL, Li[TFSI] extends the stable electrochemical window of the electrolyte at platinum by nearly 1.4 V vs Ag/Ag^+^. Similar extensions of the reducing limiting potential in lithium-IL electrolyte systems are well known and have been attributed to the breakdown of anionic species into stable solid-electrolyte interface (SEI) layers boosted by the presence of lithium ions.^11,13,41,42^ The presence of new reductive (*E*_red_ < −3.6 V vs Ag/Ag^+^) and oxidative (*E*_ox_ = −2.8 and −2.4 V) peaks, associated with the addition of Li^+^ to the electrochemical system, is characteristic of electroplating/stripping behavior of past studied ionic liquid electrolytes.^11,12^ Electrochemical reversibility for faradaic processes can be determined by analyzing the corresponding charges for both the reduction and oxidation processes, with a ratio of *Q*_red_/*Q*_ox_ approaching unity denoting electrochemical reversibility.^43^ Integration of the oxidative and reductive current reveals that the lithium electrodeposition stripping in BIL **2** does not reach reversibility (*Q*_red_/*Q*_ox_ = 1.56), favoring reductive processes over oxidations. Alternatively, the reductive current contributing to the tailing seen ca. −4 V (vs Ag/Ag^+^) is likely a combination of electroplating and the irreversible breakdown of electrolytes obscuring the true reversibility of Li^+^ within BIL systems.^12^ Regardless, the results above demonstrate how BIL electrolytes can sustain the large negative potential needed to support lithium ion electrochemistry, among other high voltage processes needed for electrochemical energy storage.

In a similar manner, cyclic voltammograms of BIL **3**, both neat and with 0.45 mol kg^–1^ Li[TFSI], were measured and compared against BIL **2** (Figure S5). Interestingly, BIL **3** exhibited a much wider electrochemical window at the platinum working electrode than neat BIL **2**, more so than what was observed at glassy carbon electrodes, demonstrating the substrate dependence of the electrochemical behavior. Upon the addition of Li[TFSI], the onset of lithium reduction occurred at potentials near −3.5 V versus Ag/Ag^+^, consistent with BIL **2**. Repeated cycling revealed a larger *Q*_red_/*Q*_ox_ for BIL **3**, an average value of 2.10 over five cycles, indicating worse reversibility over BIL **2** (Figure S6) or alternatively a higher degree of electrolyte decomposition. Despite the innate electrochemical stability of boronium electrolytes, a better understanding of the dynamics of SEI formation at the electrode surface is needed before full integration of BIL electrolytes into lithium-based energy storage.^44^ Regardless, BILs hold immense promise as materials in alternate forms of electrochemical energy storage as high voltage electrolyte substitutes for the IL and organic systems.

### Density and Viscosity of *N*-Alkylpyrrolidinium Boronium Cation-Based ILs

With similar electrochemical stabilities observed for the reported BIL electrolytes, changes in the structure can be made to improve physical properties relevant to electrolyte performance. Lowering viscosity, for instance, can directly improve electrochemical processes such as charge transport, ionic conduction, and ion diffusion.^45^ Dynamic and kinematic viscosities, along with the density, were measured for each BIL across a series of temperatures using a densitometer/viscometer (Table 3 and Figure S7).

**Table 3:** Density and Viscosity Data Were Measured at 20 °C for BILs **1**–**4**

BIL	density (g cm^–3^)	dynamic visc. (mPa s)	kinematic visc. (mm^2^ s^–1^)
(**1**) [N_112_N_112_BH_2_]	1.350	156.78	116.16
(**2**) [(1-m-pyrr)N_111_BH_2_]	1.392	130.67	93.88
(**3**) [(1-e-pyrr)N_111_BH_2_]	1.372	167.15	121.81
(**4**) [(1-m-pyrr)_2_BH_2_]	1.391	190.25	136.81

Despite differences in the molecular weight and geometry of each cation, the BIL densities were quite similar, in the range of 1.35–1.40 g cm^–3^. Dynamic viscosities at 20 °C decreased in the order BIL **4** > **3** > **1** > **2** (Table 2). As samples were heated to 30 °C, the viscosities of BILs **1**, **3**, and **4** converged to similar values, still higher than those of BIL **2** (Figure S7). Past results have shown that ILs with limited capability for charge delocalization across the cation, as is the case with tetraalkylamine groups, or longer alkyl substitutions tend to exhibit higher solution viscosities.^37,46^ At room temperature, BIL **2** exhibits a significantly lower viscosity, making it a promising candidate for electrochemical energy storage testing under ambient conditions due to enhanced ion mobility over the other species tested in this study. Upon gentle heating, up to 50 °C, the dynamic and kinematic viscosities of the suite of BILs converged around 40 mPa and 30 mm^2^ s^–1^, respectively, mitigating any loss of ionic conductivity to solution fluidity (see Figure S7).

### Ion Dynamics and Transport Properties of *N*-Alkylpyrrolidinium Boronium Cation-Based ILs

**Figure 7:**
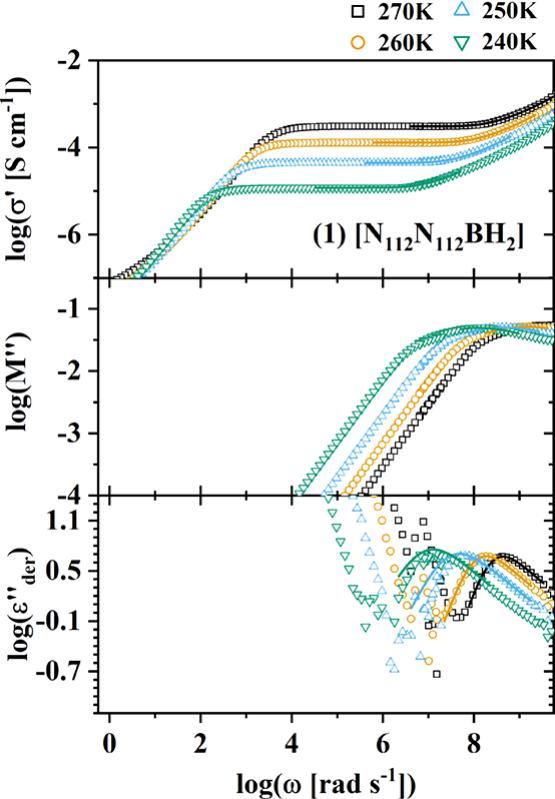
Broadband dielectric spectra of BIL **1**. Top: Real part of complex conductivity; middle: imaginary part of the complex electric modulus; bottom: derivative representation of the real part of the complex dielectric permittivity. Lines represent separate and distinct fits to each of the three representations, see eqs S1 and S2.

Broadband dielectric spectroscopy (BDS) was utilized to investigate the ion dynamics and temperature-dependent dc ionic conductivities of each BIL.^47−51^ The dielectric spectra were analyzed in the conductivity, dielectric permittivity, and electric modulus formalisms following the procedure applied in previous investigations of imidazolium ILs.^52,53^ In the provided analysis (Figure 7), the dielectric spectra of BIL **1** are presented in terms of the real part of the complex conductivity, the real part of the complex electric modulus, and the derivative representation of the real part of the complex dielectric permittivity.

**Figure 8:**
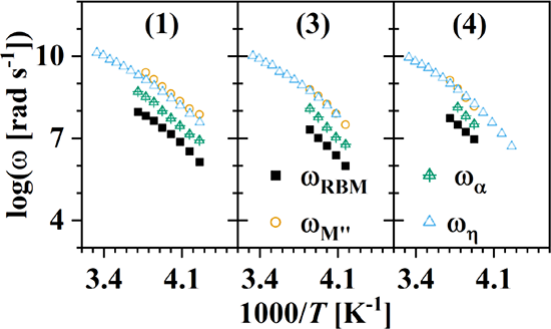
Relaxation rates of BILs **1**, **3**, and **4** were extracted from the dielectric spectra and calculated using the zero-shear fluidities.

The conductivity spectra were fit with the random barrier model (RBM), providing the value of the dc ionic conductivity, σ_0_, and the onset frequency of long-range ion transport, ω_RBM_ = 1/τ_RBM_. The modulus spectra were fit with a Havriliak–Negami function to obtain the frequency of the peak maximum, ω_M^″^_. The real part of the dielectric permittivity was fit with a separate Havriliak–Negami function, providing the rate of the primary α-relaxation, ω_α_. The dielectric spectra of BILs **3** and **4**, the fitting functions, and additional fit parameters are provided in the Supporting Information (Figures S8–S13). The relaxation rates obtained from the dielectric spectra are also provided (Figure 8).

Included in this figure are the rates of the structural relaxation, ω_η_*,* estimated from the measured fluidities (Figure S14) using Maxwell’s relation, ω_η_ = η_0_^–1^*G*_∞_, and assuming a value of the high frequency limiting glassy shear modulus, *G*_∞_ = 1 × 10^9^*Pa*.^53−55^ The data for BIL **2** are not included in this figure due to its greater tendency for crystallization, which prevented investigation of its ion dynamics by BDS.

In our prior studies, the separation in the rates extracted from the various representations, ω_RBM_, ω_M^″^_, and ω_α_, was found to be dependent upon both temperature and the chemical structure of the anion.^52,53^ The separation was attributed to a type of dynamic heterogeneity at the nearest neighbor and next nearest neighbor length scales. This attribution was supported by comparison with prior neutron-spin–echo and molecular dynamics simulations as well as the observed influence of the local dynamic heterogeneity on the dielectric signature of the motion of mesoscale solvophobic aggregates. For the boronium ILs, a comparatively greater degree of separation in the rates is observed relative to those rates obtained for the shared anion IL, 1-methyl-3-octylimidazolium bis(trifluoromethylsulfonyl)imide ([C_8_MIm][TFSI]). For BILs **1** and **3**, the fastest rates, ω_M^″^_ (Figure 8, yellow circle), and slowest rates, ω_RBM_ (Figure 8, black squares), are separated by a factor of approximately 30 while the separation is a factor of 20 for BIL **4** (compared to a factor of 8 for [C_8_MIm][TFSI]). This interesting result indicates a greater degree of local ion dynamic heterogeneity in the boronium ILs than in the corresponding imidazolium ILs. Furthermore, inspecting the peak in the imaginary electric modulus (Figure 7, middle frame), we find that there is a pronounced low-frequency shoulder for the BILs and that the height at the peak maximum is lower than that in the imidazolium ILs. These results indicate subtle differences in the local ion dynamics in the boronium-containing ILs. The overall dynamics become faster in the order BIL **1** > BIL **3** > BIL **4**. The local ion dynamics underlie and are primarily responsible for the transport properties of ILs, i.e., the dc ionic conductivities and zero-shear viscosities, which are critical factors in applying these novel materials in applications like electrochemical energy storage.^56^

To supplement analysis of the BDS response of prepared BILs, the temperature-dependent fluidity (measured from shear flow rheology) and dc ionic conductivity were measured to gain insight into the role of macromolecular rearrangements or ion mobility on charge transport. The rheological response (Figure S14) was fit using the following form of the Vogel–Fulcher–Tammann equation:
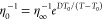
1

**Figure 9:**
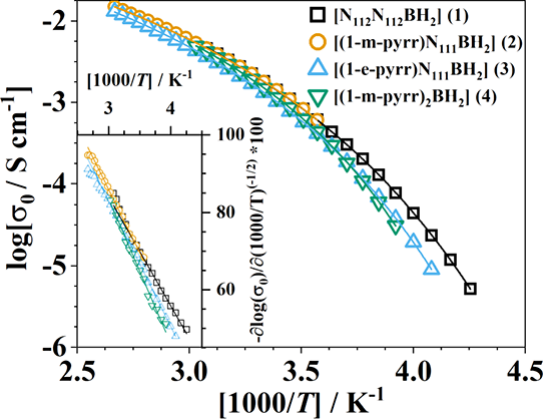
DC ionic conductivities, σ_0_, vs inverse temperature for the studied BILs. Solid lines are fits from the Vogel–Fulcher–Tammann equation. The first derivative of the conductivity is shown in the inset plot.

In a similar manner, the temperature-dependent dc ionic conductivity (Figure 9) for each BIL was fit by using the equation below:
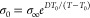
2

Relevant parameters extracted from the fit of both sets of data (*D*, *T*_0_, η_∞_^–1^, and σ_∞_) are provided in Table S1. It is worth noting that the room temperature conductivity of each BIL, regardless of electrochemical performance and slight differences in viscosity, converges around 1 × 10^–3^ S cm^–1^, which is comparable to commonly studied TFSI-containing ILs.^57^ Further analysis of the temperature dependence of the first derivative of the DC ionic conductivity (Stickel analysis) reveals a simple linear trend across all temperatures for each sample (Figure 9, inset).

The first derivative plot of the ionic conductivities of ILs typically yields straight lines with uniform slopes across the measured temperature range, indicating a single VFT temperature dependence.^53,58^ In instances where two or more regions with different slopes are observed, multiple VFT functions are needed to fully depend on conductivity.^59^ These factors, along with the physical characterization of the BIL materials provided above, contribute to the ability of BILs to serve as a class of electrolyte and should help inform initial probes into their use in battery and supercapacitor devices.

## Conclusions

In this work, a new series of boronium ILs with promising thermal and electrochemical properties are reported. Of the cations reported, electrochemical windows of 6.3 and 6.1 V were achieved with BILs **3** and **2**, respectively, highlighting the electrochemical impact of adding a single *N*-alkylpyrrolidinium moiety to the cation. Furthermore, BIL **2** ([(1-m-pyrr)N_111_BH_2_][TFSI]), one of the top-performing ionic liquids in this study, demonstrated the capacity for the electrochemical plating and stripping of lithium. Preliminary BDS measurements of the BILs reveal a greater degree of separation in the rates associated with local ion dynamics, which determine ionic conductivity, when compared to imidazolium-based ILs. Such impressive electrochemical and physical properties could be further improved by exchanging the TFSI anion with more stable species like [PF_6_]^−^, further expanding their potential for use in diverse electrochemical applications.

## Supporting Information Available

Please see the Supporting Information for a complete experimental section detailing the synthesis and characterization of each BIL. This material is available free of charge at the Web site: https://pubs.acs.org. The Supporting Information is available free of charge at https://pubs.acs.org/doi/10.1021/acs.inorgchem.3c02971.

Additional figures provided such as plots of the thermogravimetric analysis, additional electrochemical analysis of Li-containing samples of BIL, and complete workup of the dielectric response for BILs **3** and **4** (PDF)
