# Supplementary material for: Physical and Electrochemical Analysis of N-Alkylpyrrolidinium-Substituted Boronium Ionic Liquids
Source: Inorg Chem. 2023 Oct 23;62(44):18280–9. doi: 10.1021/acs.inorgchem.3c02971 (PMC10630938; doi:10.1021/acs.inorgchem.3c02971)
Supplement: Supplementary file 1 — ic3c02971_si_002.pdf [file ic3c02971_si_002.pdf]

Supporting Information

Physical and Electrochemical Analysis of N-Alkyl Pyrrolidinium Substituted Boronium Ionic Liquids

Christopher. D. Stachurski[1*], James. H. Davis Jr.[2*], Tyler Cosby[3], Margaret E. Crowley[2], Nathaniel E. Larm[1], Mollie G. Ballentine[2], Richard A. O'Brien[2], Matthias Zeller[4], E. Alan Salter[2], Andrzej Wierzbicki[2], Paul C. Trulove[1], and David. P. Durkin[1*]

[1] Department of Chemistry, U. S. Naval Academy, Annapolis, Maryland 21402, USA
[2] Department of Chemistry, University of South Alabama, Mobile, Alabama 36688, USA
[3] School of Mathematics and Sciences, University of Tennessee Southern, Pulaski, Tennessee 38478, USA
[4] Department of Chemistry, Purdue University, West Lafayette, Indiana, 47907, USA

*** Corresponding: Dr. Christopher D. Stachurski (stachurs@usna.edu), Dr. James H. Davis, (jdavis@southalabama.edu), Dr. David P. Durkin (durkin@usna.edu)**

Additional Experimental Details

Synthetic Protocol and NMR Characterization

The chemicals dimethylsulfide-borane, trimethylamine-borane, *N*,*N*-dimethylethylamine (>99%), *N*-methylpyrrolidine (>98% GC), and iodine (>99.8%) were purchased from SigmaAldrich and used as received. *N*-ethylpyrrolidine (96%) was purchased from Combi-Blocks and also used as received. Potassium bis(trifluoromethanesulfonyl)imide K[TFSI] was prepared by the neutralization of 80% aqueous bis(trifluoromethanesulfonyl)imidic acid using reagent grade KOH, followed by crystallization from water and drying under vacuum. All solvents were ACS reagent grade. Deuterated solvents utilized for NMR spectroscopy were purchased from Cambridge Isotope Laboratories and used as received. All manipulations were conducted in air, but inside a well-ventilated fume hood due to the generation of H_2 gas, dimethylsulfide, and the volatile, malodorous character of the amines.

Synthesis of IL **1**. A 1 L Erlenmeyer flask was charged with a magnetic stirring bar and 500 mL of toluene. Via syringe, 35.0 g (28.0 mL, 0.46 mol) of dimethylsulfide borane was added to the toluene while stirring. To the vigorously stirred, clear, colorless solution was added (over approximately 10 min) 58.3 g (0.23 mol) of crystalline I_2. Each addition engendered vigorous gas evolution, and the solution remained clear but became deep brown-red in color [Caution: Adding the I_2 too fast can lead to overflow by frothing of the solution]. Once the iodine addition was complete, stirring was continued for thirty minutes, during which time fading of the solution color occurred. Then, while the solution was being stirred vigorously, 67.3 g (0.92 mol) of dimethylethylamine was added in one portion. The solution color rapidly faded to light yellow, and within minutes a white solid began to precipitate. Stirring was then continued for 24 h, although visually the reaction appeared to have been complete within just a few hours. Stirring

was stopped, and the pale yellow-white precipitate was separated from the liquid by vacuum filtration using a Buchner funnel. While still on the funnel, the solid was washed with two 50 mL portions of toluene, followed by one 50 mL portion of diethylether. The solid was transferred to a round-bottomed flask and dried overnight by rotary evaporation. Yield: 109.2 g (83%). Formation of the desired cation was verified by ^{1}H-, ^{13}C-, and ^{11}B-NMR.

Next, 50.0 g (0.17 mol) of the iodide salt (above) was dissolved in 250 mL of water. While stirring, 64 g of K[TFSI] was added in portions. As the latter dissolved, the solution became cloudy, and as addition continued a clearly defined, pale-yellow second liquid phase became apparent. After stirring overnight, stirring was stopped and the phases were allowed to separate. The lower (product) phase was then washed and separated twice using 100 mL portions of water, after which it was dissolved in CH_2Cl_2, and anhydrous $MgSO_4$ was added to dry the solution. After removing the $MgSO_4$ by filtration, the CH_2Cl_2 was removed with a rotary evaporator. The remaining clear, colorless ionic liquid was then dried for 12 h at 10 mbar while being heated to 50 °C. Accounting for slight mass losses from transferring the liquid product **1**, the yield of the ion metathesis step was quantitative. **NMR** (Figure S15-17) (500 mHz, DMSO-d^{6}). ^{1}H: δ 1.17 (t, 6H, *CH_3*-CH_2), δ 1.9 (very broad, *B-H*), δ 2.66 (s, 12H, N-*CH_3*), δ 3.04 (4H, CH_3-*CH_2*). ^{13}C: δ 8.25 (*CH_3*-CH_2), δ 48.8 (CH_3-*CH_2*), δ 57.8 (N-*CH_3*), δ 119.5 (q, -*CF_3*). ^{10}B: δ 1.18.

Synthesis of IL **2**. A 1 L Erlenmeyer flask was charged with a magnetic stirring bar and 500 mL of toluene. To the stirred toluene was added 50.0 g (0.68 mol) of trimethylamine borane was added in one portion. To the vigorously stirred, clear, colorless solution was then added (over approximately 10 min) 86.9 g (0.34 mol) of crystalline I_2. Each addition engendered vigorous gas evolution, and the solution remained clear but became deep brown in color. Once the iodine addition was complete, stirring was continued for thirty minutes, during which time the solution

faded to pale yellow. Then, while the solution was being stirred vigorously, 58.3 g (0.68 mol) of *N*-methylpyrrolidine was added in one portion. The solution color rapidly faded to light yellow, and within minutes a white solid began to precipitate. Stirring was then continued for 24 h, stopped, and the white precipitate separated from the liquid by vacuum filtration using a Buchner funnel. While still on the funnel, the solid was washed with two 50 mL portions of toluene, followed by one 50 mL portion of diethylether. The solid was transferred to a round-bottomed flask and dried overnight by rotary evaporation. Yield: 177 g (91%). The identity of the product was verified by ^{1}H-, ^{13}C-, and ^{11}B-NMR.

In the next step, 50.0 g (0.18 mol) of the iodide salt (above) was dissolved in 250 mL of water. While stirring, 60 g of K[TFSI] was added in small portions. As the latter dissolved, the solution became cloudy, and as addition continued a colorless, well defined, second liquid phase became apparent. After stirring overnight, stirring was stopped and the phases allowed to separate. The lower (product) phase was then washed and separated twice using 100 mL portions of water, after which it was dissolved in CH_2Cl_2, and anhydrous $MgSO_4$ added to dry the solution. After removing the $MgSO_4$ by filtration the CH_2Cl_2 was removed with a rotary evaporator. The remaining clear, colorless ionic liquid was then dried for 12 h at 10 mbar while being heated to 50 °C. Considering small mass losses from transferring the liquid product **2**, the yield from the ion metathesis step was quantitative. **NMR** (Figure S18-S20) (500 mHz, DMSO-d^6). ^{1}H: δ 1.93 (complex m, 2H, N-CH_2-$\mathit{CH_2}$), δ 1.98 (complex m, 2H, N-CH_2-$\mathit{CH_2}$), δ 2.75 (s, 3H, N-$\mathit{CH_3}$), δ 2.98 (complex m, 2H, N-$\mathit{CH_2}$-CH_2), δ 3.25 (complex m, 2H, N-$\mathit{CH_2}$-CH_2), δ 3.50 (s, 3H, *N*-$\mathit{CH_3}$) ^{13}C: δ 20.9 (N-CH_2-$\mathit{CH_2}$), δ 46.2 (N-$\mathit{CH_2}$), δ 52.1 (N-$\mathit{CH_3}$), δ 61.7 (N-$\mathit{CH_3}$), δ 119.5 (q, -$\mathit{CF_3}$). ^{10}B: δ 1.05.

Compound **2**, BPh_4 salt. In a 100 mL Erlenmeyer flask charged with a stir bar, 2.5 g (8.8 mmol) of the iodide salt of **2** was dissolved in 30 mL of water. In a separate flask, 3.4 g (10 mmol) of $NaBPh_4$ was dissolved in 50 mL of warm water. While stirring, the latter solution was slowly added to that containing the iodide salt of **2**. A voluminous white precipitate formed immediately. After stirring for an additional 30 min, the solid and supernatant were separated using a Buchner funnel, and the white solid product washed twice with 10 mL of warm water. After air drying, the solid was dissolved in a minimal volume of boiling acetone-methanol (c.a. 50/50 v/v) and put aside. Upon cooling and slow evaporation, crystals suitable for single-crystal analysis were obtained.

Synthesis of IL **3**. A 1 L Erlenmeyer flask was charged with a magnetic stirring bar and 500 mL of toluene. To the stirred toluene was added 50.0 g (0.68 mol) of trimethylamine borane was added in one portion. To the vigorously stirred, clear, colorless solution was then added (over approximately 10 min) 87.0 g (0.34 mol) of crystalline I_2. Each addition engendered vigorous gas evolution, and the solution remained clear but became deep brown in color. Once the iodine addition was complete, stirring was continued for thirty minutes, during which time the solution faded to pale yellow-orange. Then, while the solution was being stirred vigorously, 68.0 g (0.68 mol) of *N*-ethylpyrrolidine was added in one portion. The solution color rapidly faded to light colorless; over the course of several hours, white solid began to precipitate. Stirring was then continued for 48 h, stopped, and the ivory-colored precipitate separated from the liquid by vacuum filtration using a Buchner funnel. While on the funnel, the solid was washed with two 50 mL portions of toluene, followed by one 50 mL portion of diethylether. The solid was transferred to a round-bottomed flask and dried overnight by rotary evaporation. Yield: 157 g (77%). The identity of the product was verified by ^{1}H-, ^{13}C-, and ^{11}B-NMR.

In the next step, 50.0 g (0.17 mol) of the iodide salt (above) was dissolved in 250 mL of water. While stirring, 60 g of K[TFSI] was added in small portions. As the latter dissolved, the solution became cloudy, and as addition continued a near-colorless, well defined, second liquid phase became apparent. After stirring overnight, stirring was stopped and the phases allowed to separate. The lower (product) phase was then washed and separated twice using 100 mL portions of water, after which it was dissolved in CH_2Cl_2, and anhydrous $MgSO_4$ added to dry the solution. After removing the $MgSO_4$ by filtration the CH_2Cl_2 was removed with a rotary evaporator. The remaining clear, colorless ionic liquid was then dried for 12 h at 10 mbar while being heated to 50° C. As in the case of the previously described syntheses, the yield of **3** from the ion metathesis step was essentially quantitative. **NMR** (Figure S21-S23) (500 mHz, acetone-d^6). ^{1}H, δ: 1.30 (t, 3H, CH_2-CH_3); 2.86 (s, 9H, N-CH3); 2.88 (multiplet, partially obscured by adventitious H_2O and N-CH_3 peaks, assigned as N-CH_2-CH_2); 3.13 (q, 2H, N-CH_2-CH_3); 3.16 (m, 2H, -CH_2-CH_2-); 3.35 (m, 2H, -CH_2-CH_2-)[Hs of the former CH_2 groups on opposite ring faces]. ^{13}C, δ: 9.62 (CH_2-CH_3); 22.27 (-CH_2-CH_2-); 51.67 (N-CH_2-CH_3); 52.79 (N-CH_3); 60.15 (N-CH_2-CH_2-); 119.2 (q, CF_3); ^{11}B, δ: -2.63.

<u>Synthesis of ionic liquid **4**.</u> The procedure for the synthesis of IL **4** followed that of **1**, but the stirring time after the addition of the *N*-methylpyrrolidine to the intermediate iodoborane was increased to 72 h. The following quantities of materials were used: Dimethylsulfide-borane, 25.0 g (20.0 mL, 0.33 mol); iodine, 41.8 g (0.16 mol); *N*-methylpyrrolidine, 56.2 g (0.66 mol); Yield of iodide salt: 63.5 g (0.20 mol, 62%). 50.0 grams of the product iodide salt (0.16 mol) were converted to **4** using 57.5 g (0.18 mol) K[TFSI]. Yield of **4**: 81 g (0.17 mol, 98%). **NMR** (Figure S24-S26) (500 mHz, DMSO-d^6). ^{1}H: δ 1.89 (complex m, 4H, N-CH_2-CH_2), δ 1.98 (complex m, 4H, N-CH_2-CH_2), δ 2.75 (s, 6H, N-CH_3), δ 2.98 (complex m, 4H, N-CH_2-CH_2), δ 3.25 (complex

m, 4H, N-CH_2-CH_2). ^{13}C: δ 20.7 (N-CH_2-CH_2), δ 46.1 (N-CH_2), δ 61.3 (N-CH_3), δ 119.5 (q, -CF_3). ^{10}B: δ 2.25.

Compound **4**, BPh_4 salt. In a 100 mL Erlenmeyer flask charged with a stir bar, 2.2 g (7.0 mmol) of the iodide salt of **4** was dissolved in 25 mL of water. In a separate flask, 2.6 g (7.7 mmol) of $NaBPh_4$ was dissolved in 50 mL of warm water. While stirring, the latter solution was slowly added to that containing the iodide salt of **4**. A voluminous white precipitate formed immediately. After stirring for an additional 30 min, the solid and supernatant were separated using a Buchner funnel, and the white solid product washed twice with 10 mL of warm water. After air drying, the solid was dissolved in a minimal volume of boiling acetone-methanol (c.a. 50/50 v/v) and put aside. Upon cooling and slow evaporation, crystals suitable for single-crystal analysis were obtained.

Thermal Characterization

Thermal Gravimetric Analysis

The thermal stability of each BIL was evaluated using thermal gravimetric analysis (TGA) (TA Instruments Q500). Samples were loaded onto platinum pans and evaluated under nitrogen from 20-900 °C at a heating rate of 10 °C min^{-1}. The decomposition temperature (T_5) was taken as the point at which 5 wt% of the initial sample mass was lost.

Differential Scanning Calorimetry

Thermal transitions were measured using differential scanning calorimetry (DSC) (TA Instruments Q2000) equipped with liquid nitrogen cooling. Each BIL was sealed in Tzero hermetic aluminum pans in a nitrogen filled glovebox and analyzed first by heating to 50 °C, then cycled

twice between -150 °C and 50 °C at 10 °C min^{-1} under a helium purge at a flow rate of 25 mL min^{-1}. Glass transition temperatures (T_g) were measured from the heating curves for each BIL.

Single Crystal Structure Determinations

Crystal structures of the BPh_4 salts of BIL **2** and **4** were determined by single crystal X-ray diffraction using a Bruker Quest diffractometer with a fixed chi angle, a Mo Kα wavelength (λ = 0.71073 Å) sealed tube fine focus X-ray tube, single crystal curved graphite incident beam monochromator, a Photon II area detector, and an Oxford Cryosystems low temperature device. Data were collected at 150 K, reflections were indexed and processed, and the files scaled and corrected for absorption using APEX3[1] and SADABS[2]. The space groups were assigned using XPREP[3-4] and solved by direct or dual methods using ShelXS[4] or ShelXT[5] and refined by full matrix least squares against F^2 with all reflections using Shelxl2018[6] with the graphical interface Shelxle[7]. H atoms attached to carbon and boron atoms were positioned geometrically and constrained to ride on their parent atoms. C-H bond distances were constrained to 0.95 Å for aromatic C-H moieties, and to 0.99 and 0.98 Å for aliphatic CH_2 and CH_3 moieties, respectively. B-H bond distances were allowed to refine. Methyl CH_3 were allowed to rotate but not to tip to best fit the experimental electron density. $U_{iso}(H)$ values were set to a multiple of $U_{eq}(C)$ with 1.5 for CH_3 and 1.2 for all other H atoms. BIL**4**-BPh_4 was refined as a 2-component merohedric twin (high/low symmetry hexagonal, 180° rotation around (0 1 0)). Application of the 3×3 transformation matrix 0 1 0 1 0 0 0 0 -1 yielded a minor twin fraction of 0.453(2).

In BIL**2**-BPh_4 the cation shows minor disorder by an approximate 180° rotation, exchanging the trimethylamine and *N*-methylpyrrolidine fragments with each other. Also disordered is one

phenyl group of the anion, in the proximity of the disordered cation. The phenyl disorder is likely induced by the cation disorder, but occupancies were refined independently from each other. For both disordered fragments the major and minor moieties were restrained to have similar geometries. The major and minor B-C(phenyl) bond lengths were restrained to be similar to each other. U_{ij} components of ADPs for disordered atoms closer to each other than 2.0 Å were restrained to be similar. Subject to these conditions the occupancy ratio refined to 0.915(2) to 0.085(2) for the cation, and to 0.871(3) to 0.129(3) for the phenyl group. The lengths of the B-H bonds were refined but restrained to be similar to each other.

Electrochemical Characterization

Electrochemical Measurements

All electrochemical experiments were performed in a nitrogen filled glovebox (<1 ppm H_2O). Cyclic voltammetry (CV) was performed using a Biologic SP-200 potentiostat. All samples were analyzed using a three-electrode setup consisting of either a glassy carbon or a platinum working electrode (EDAQ, surface area = 7.8 x 10^{-3} cm^2), a platinum mesh counter electrode, and a home-built Ag/Ag^+ reference electrode made from a 100 mM solution of silver triflate dissolved in [EMI][TFSI] contained within a glass tube sealed with a vycor glass frit. The working and counter electrodes were held in the same compartment, not separated by a frit or membrane. Following assembly, the reference electrode was allowed to equilibrate for 24 h before use. Working electrodes were polished prior to and between uses with a series of alumina slurries (6, 1, 0.25 μm) and cleaned with a microfiber polishing pad between successive scans of the same BIL system. Prior to electrochemical analysis, BILs were first dried under vacuum with mild heating prior to storage in a nitrogen-filled glovebox to minimize the impact of water. Evaluation of the electrochemical reversibility of lithium ions at a platinum working electrode was performed

in select BIL electrolytes containing 0.45 mol kg^{-1} Li[TFSI] which were filtered using a 0.45 µm PTFE filter.

Broadband Dielectric Spectroscopy

Broadband dielectric spectroscopy (BDS) measurements were made over a range of frequencies (10^{-1} – 10^{9} Hz). A Novocontrol α-analyzer with a gold-coated brass parallel plate capacitor geometry (20 mm diameter, 1.0 mm sample thickness maintained by Teflon spacers) was used over the frequency range 10^{-1} – $3x10^{6}$ Hz. Frequencies between 10^{6} - 10^{9} Hz were measured using an HP E4991B impedance analyzer with Novocontrol RF extension and a gold-coated parallel plate capacitor (10 mm diameter, 0.1 mm sample thickness maintained by silica rod spacers). Temperature control was maintained using a Quatro temperature control system with an accuracy ±0.1 °C, using nitrogen as both a heating and cooling gas.

The dielectric spectra are analyzed in three separate representations (Figures S7-S13). The real part of complex conductivity, $\sigma^*(\omega) = \sigma^*(\omega) + i\sigma^*(\omega)$, is well described by the Random Barrier Model, **Eq. S1**.

$$\sigma^*(\omega) = \sigma_0 \left[\frac{i\omega\tau_{RBM}}{\ln(1+i\omega\tau_{RBM}}\right] \tag{S1}$$

The frequency of the peak maximum in the imaginary part of complex electric modulus, $M^* = M'(\omega) + iM''(\omega)$, is obtained by fitting with a Havriliak-Negami fitting function. The real part of the complex dielectric permittivity, $\varepsilon^*(\omega) = \varepsilon'(\omega) - \varepsilon''(\omega)$, is also fit using a Havriliak-Negami function, **Eq. S2**.

$$\varepsilon^*(\omega) = \varepsilon_\infty + \frac{\Delta\varepsilon_\alpha}{\left(1+\left(i\omega\tau_{HN,\alpha}\right)^{\beta}\right)^{\gamma}} \tag{S2}$$

The real part of complex dielectric permittivity is presented in terms of the derivative representation, $\varepsilon''_{der} = \left(-\frac{\pi}{2}\right)\frac{\partial\varepsilon'}{\partial \ln(f)}$.

Quantum-based Computations

The BIL cations **2-4** (Figure 1) and other cations of interest, along with their reduced daughter species (charge = 0, multiplicity = 2), were optimized using the Gaussian16 suite of programs[8] with the wB97X-D density functional[9] and the cc-pvtz basis set.[10] Analytic vibrational frequencies were computed to confirm optimized geometries as stable minima and to provide enthalpy-based estimates of reduction potentials (E^0_{red}) in the gas phase at 298 K.[11] Electrostatic potential maps (elstats) were computed as follow-up single-point calculations using Spartan'20.[12]

Density & Viscosity Measurements

Rheology

The temperature dependent zero-shear viscosities were measured using a Discovery HR-2 Rheometer (TA Instruments) with 25 mm stainless steel parallel plates and stress/rate-controlled flow experiments. The rheometer was contained within a home-built nitrogen-purged glovebox to maintain low moisture content (<100 ppm). Temperature control was maintained using an environmental test chamber accurate to ± 0.1 °C with nitrogen as both the heating and cooling gas.

Densitometry & Viscosity

Temperature dependent densities and kinematic-dynamic viscosities were measured using an SVM 3001 Stabinger viscometer (Anton Paar) with temperature controlling capabilities. Samples were loaded directly into the instrument prior to analysis to minimize exposure to ambient moisture.

Supplemental Tables and Figures

Table S1. Parameters for the VFT fit of dc ionic conductivities, corresponding to the solid lines in **Figures S14** and **8**.

IL	D (Conductivity)	T_0 [K] (Conductivity)	σ_∞ (S cm^{-1})	D (Fluidity)	T_0 [K] (Fluidity)	η_0^{-1} (10^4 Pa^{-1} s^{-1})
(1) [$N_{112}N_{112}BH_2$]	4.8	167	0.8	7	151	2.6
(2) [(1-m-pyrr)$N_{111}BH_2$]	3.9	177	0.5	2	198	0.1
(3) [(1-e-pyrr)$N_{111}BH_2$]	3.9	180	0.5	5	169	0.9
(4) [(1-m-pyrr)$_2BH_2$]	3.1	191	0.4	5	173	1.1

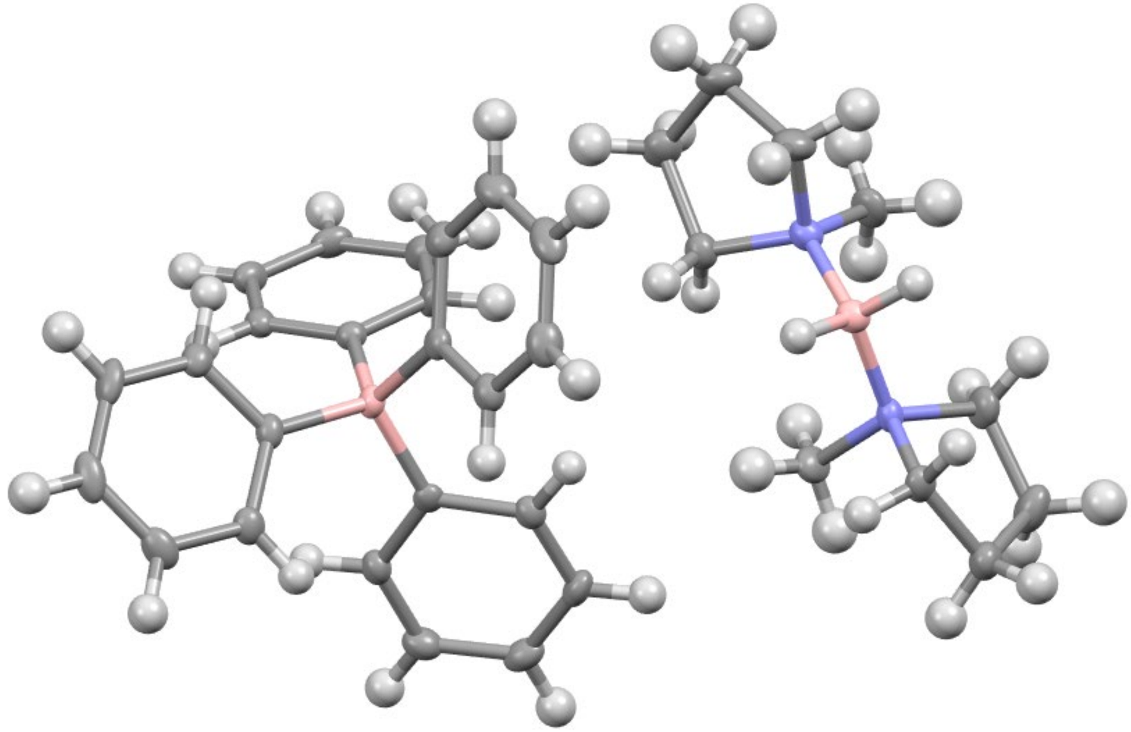

Figure S1. Ball-and-stick plot of $[(1\text{-m-pyrr})_2BH_2][BPh_4]$. In the cation of BIL **4**, clearly showing the coordination to the boron center of the two supporting *N*-methylpyrrolidine ligands.

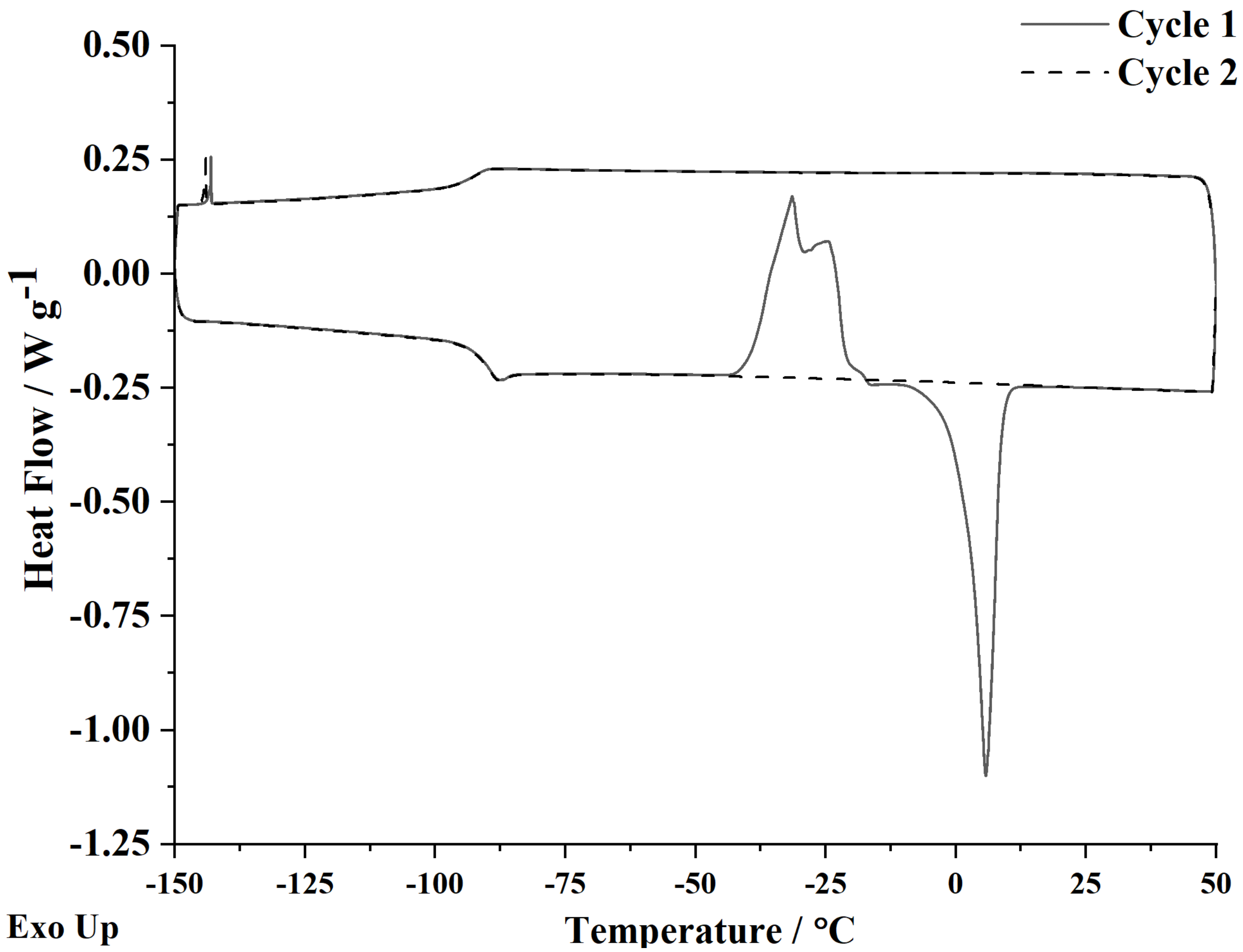

Figure S2. Differential scanning calorimetry (DSC) of BIL **1**, showing repeated thermal cycling of $[N_{112}N_{112}BH_2][TFSI]$ from 50 °C to -150 °C at a heat/cool ramp of 10 °C min^{-1}.

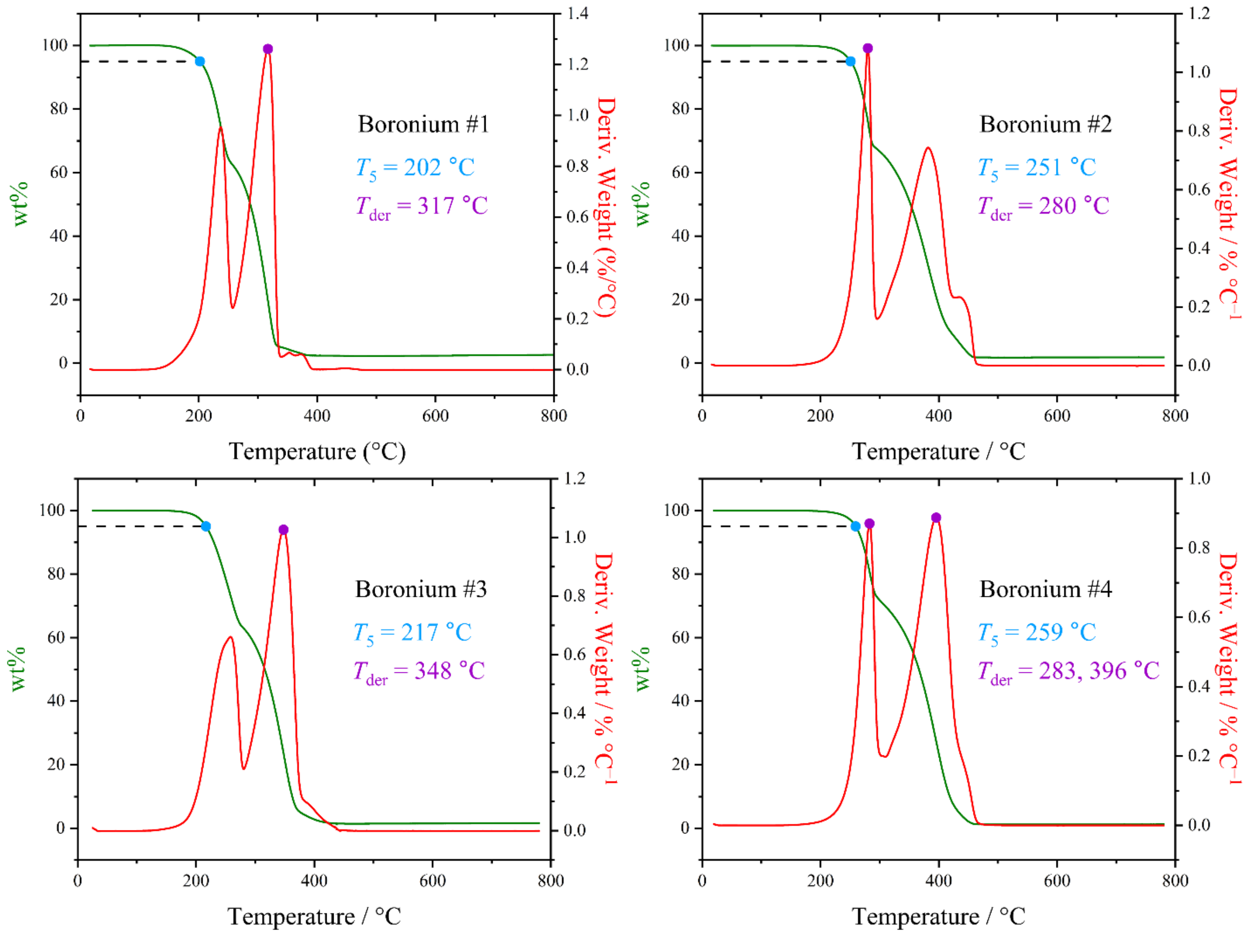

Figure S3. Thermal gravimetric analysis of BIL **1-4**. Samples were heated under nitrogen at a ramp rate of 10 °C min^{-1}. T_5 was determined as the point at which 5 wt% of the initial sample mass was lost. The first derivatives are also shown for each decomposition curve highlighting the two-step decomposition observed for each sample.

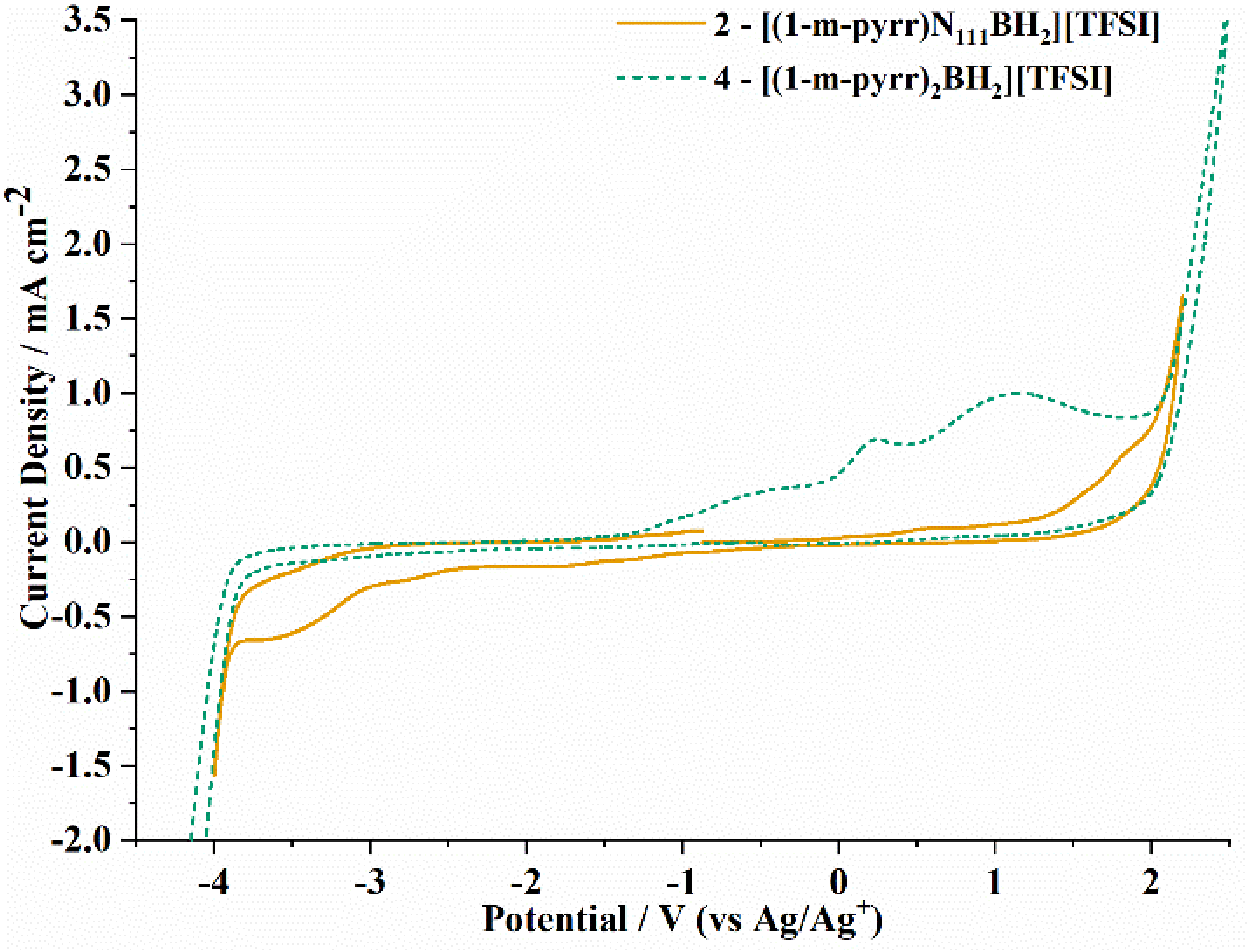

Figure S4. Cyclic voltammograms at a glassy carbon working electrode comparing the electrochemical window of [(1-m-pyrr)$N_{111}BH_2$][TFSI] (**2**) and [(1-m-pyrr)$_2BH_2$][TFSI] (**4**). Substitution of the second trialkylamine ligand for a *N*-methylpyrrolidinium moiety did not further improve the electrochemical stability of the resulting IL, unlike the what was observed between BIL **1** and **2**.

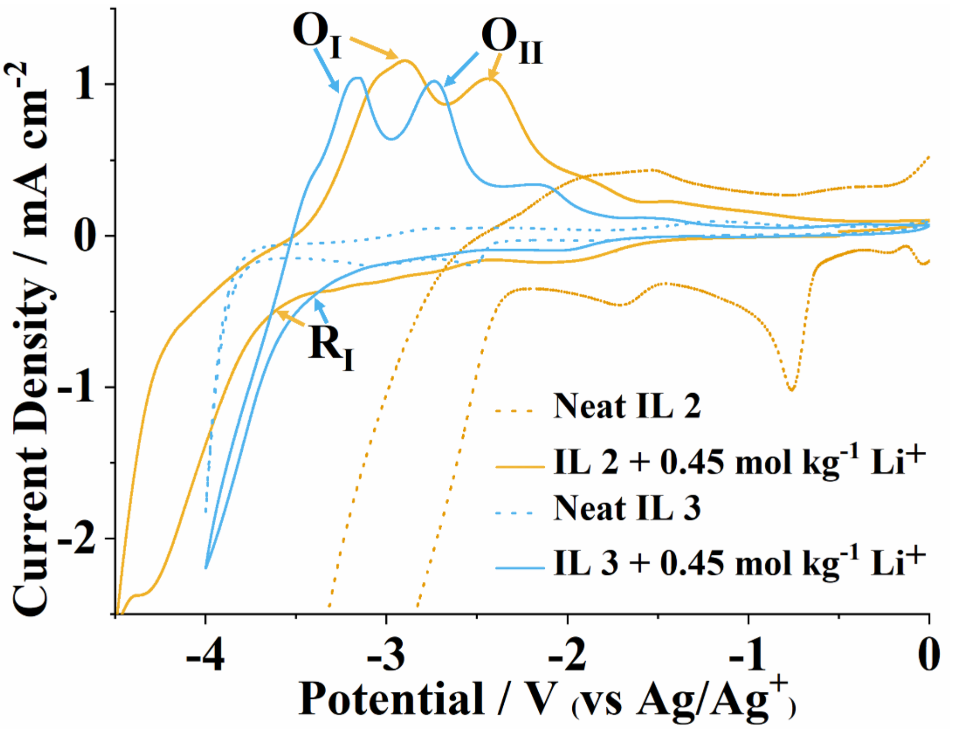

Figure S5. Cyclic voltammogram comparing lithium electroplating and stripping of [(1-m-pyrr)$N_{111}BH_2$][TFSI] (**2**) and [(1-e-pyrr)$N_{111}BH_2$][TFSI] (**3**). Cycling begun at 0 V (vs Ag/Ag^+) before sweeping positive to predetermined switching potentials based on preliminary scans identifying either the onset of Li^+ reduction or electrolyte degradation. Similar onset potential for lithium reduction were observed regardless of cation (**R_I**), though BIL **3** exhibited higher stability at a platinum working electrode than BIL **2**. Upon reserving the scan direction, two oxidation events (**O_I** and **O_{II}**) occurred in rapid succession, suggesting a primary oxidation of lithium metal is required (**O_I**) prior to oxidative stripping from the electrode surface (**O_{II}**).

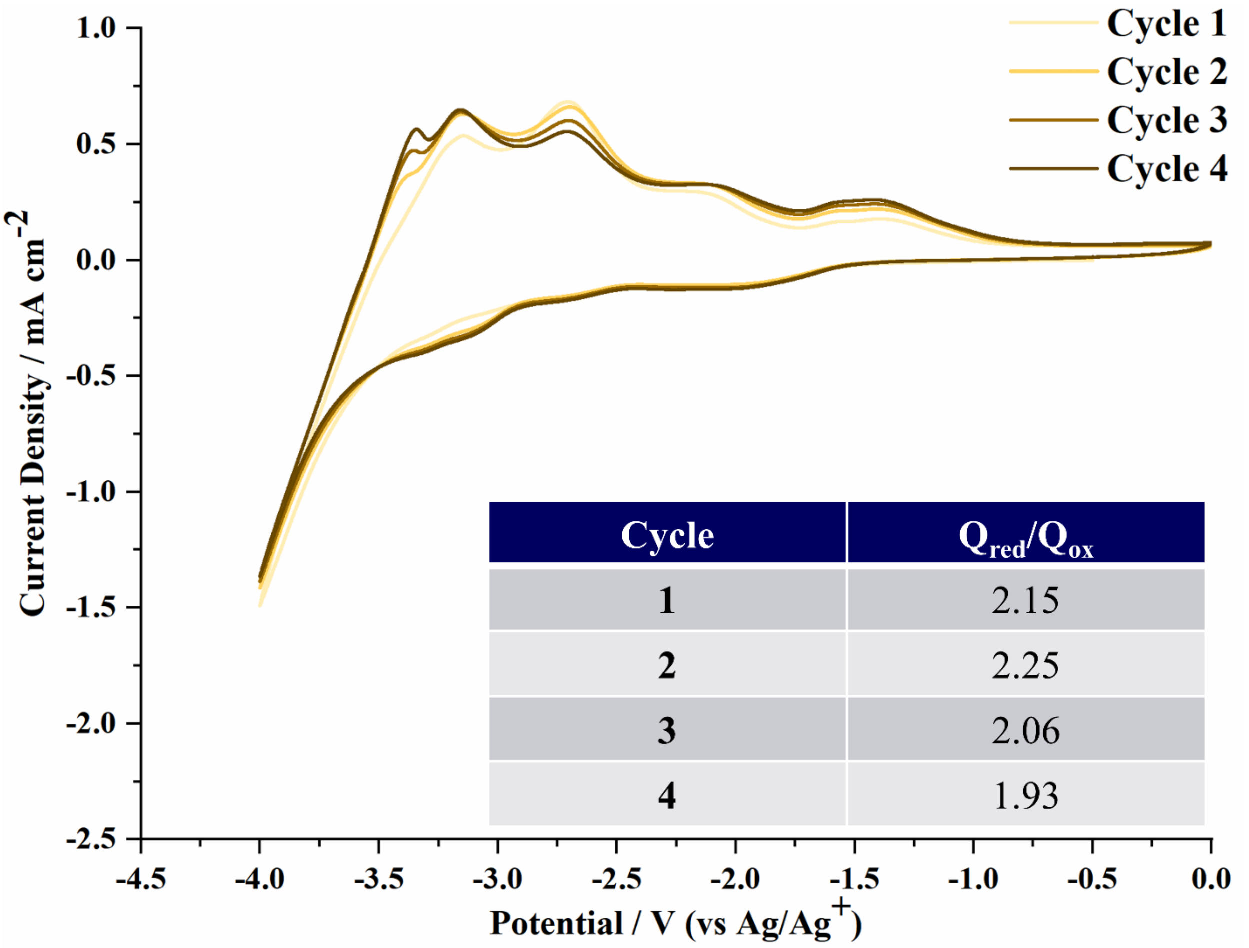

Cycle	Q_{red}/Q_{ox}
**1**	2.15
**2**	2.25
**3**	2.06
**4**	1.93

Figure S6. Cyclic voltammograms of [(1-e-pyrr)$N_{111}BH_2$][TFSI] (**3**) in the presence of 0.45 mol kg^{-1} Li[TFSI]. Continuous cycling was performed and the first four cycles are shown. Overall consistent behavior was seen across the four cycles. The electrochemical reversibility of each cycle is also provided in the inset table.

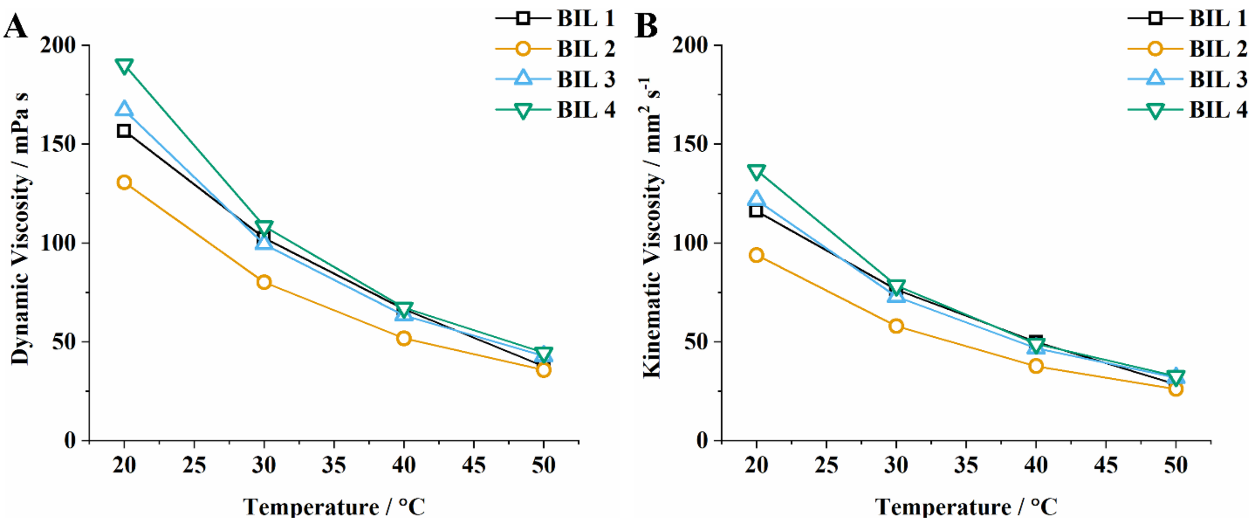

Figure S7. Temperature-dependent A) dynamic and B) kinematic viscosities for BIL **1-4**.

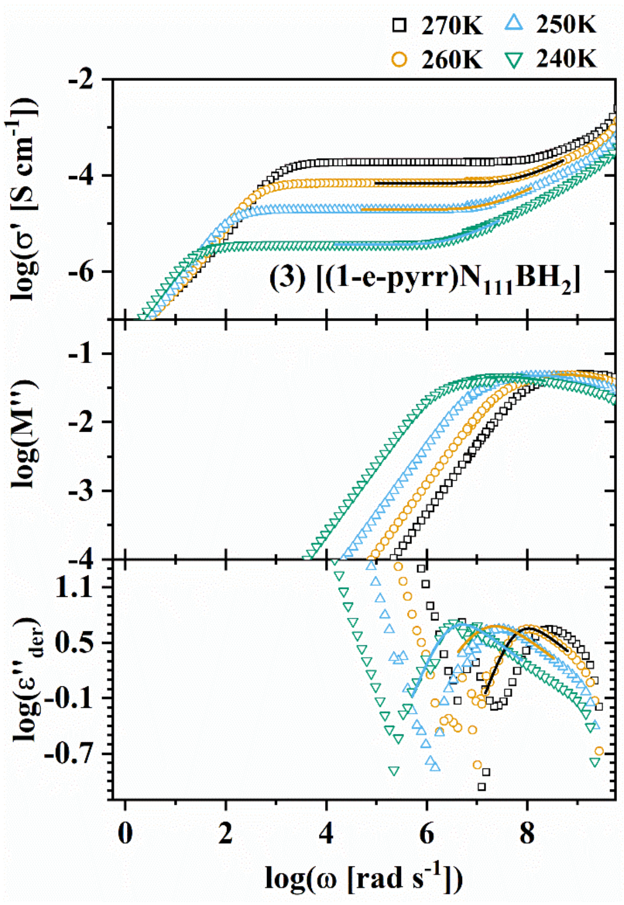

Figure S8. Broadband dielectric spectra of BIL **3**. Top: Real part of complex conductivity, Middle: imaginary part of complex electric modulus, Bottom: Derivative representation of the real part of complex dielectric permittivity. Lines represent separate and distinct fits to each of the three representations.

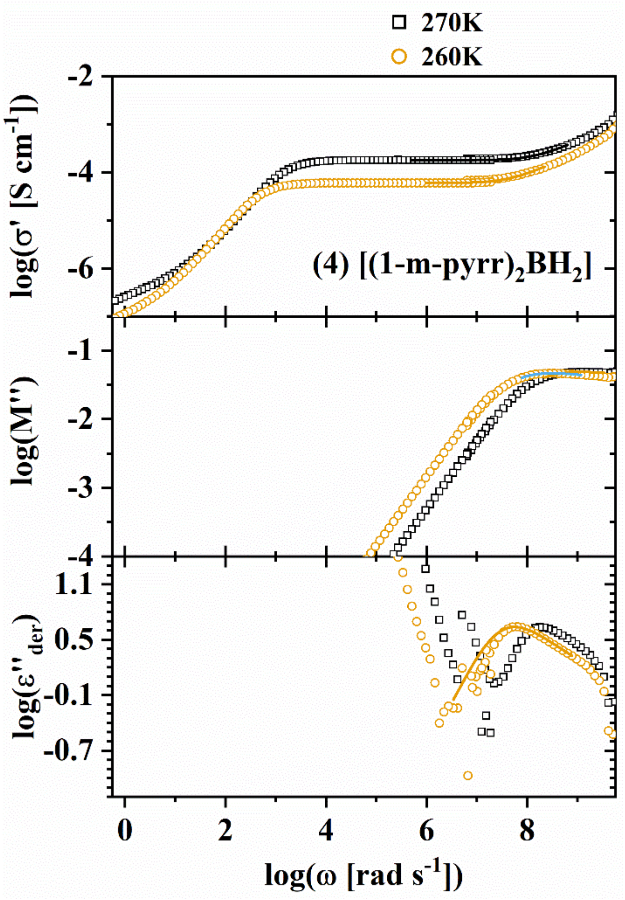

Figure S9. Broadband dielectric spectra of BIL **4**. Top: Real part of complex conductivity, Middle: imaginary part of complex electric modulus, Bottom: Derivative representation of the real part of complex dielectric permittivity. Lines represent separate and distinct fits to each of the three representations.

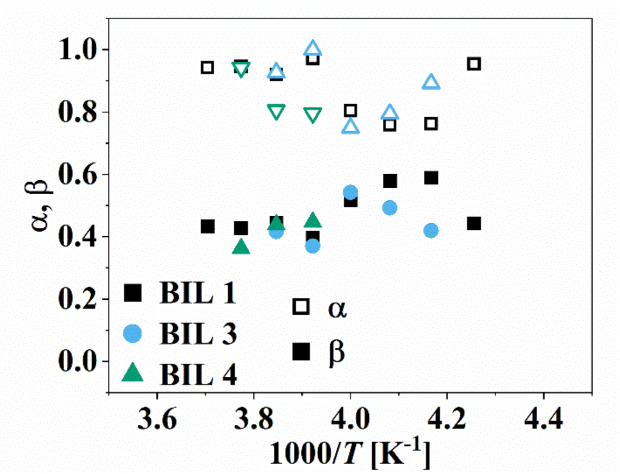

Figure S10. Shape parameters of the Havriliak-Negami fit function applied to the real part of complex dielectric permittivity. Open symbols = α, closed symbols = β.

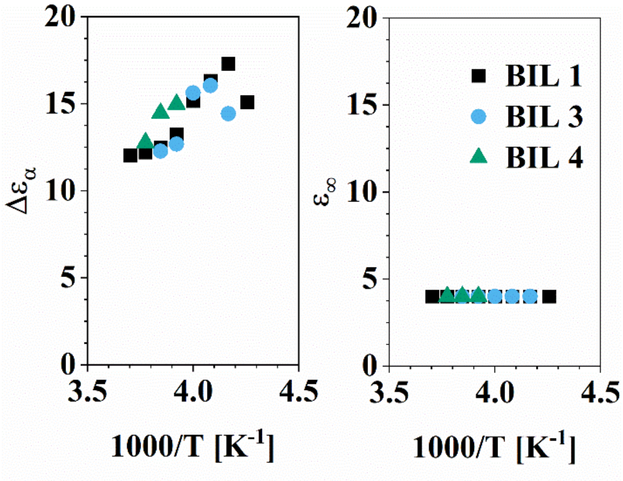

Figure S11. Dielectric strengths of the Havriliak-Negami fit function applied to the real part of complex dielectric permittivity for BILs **1**,**3** and **4**.

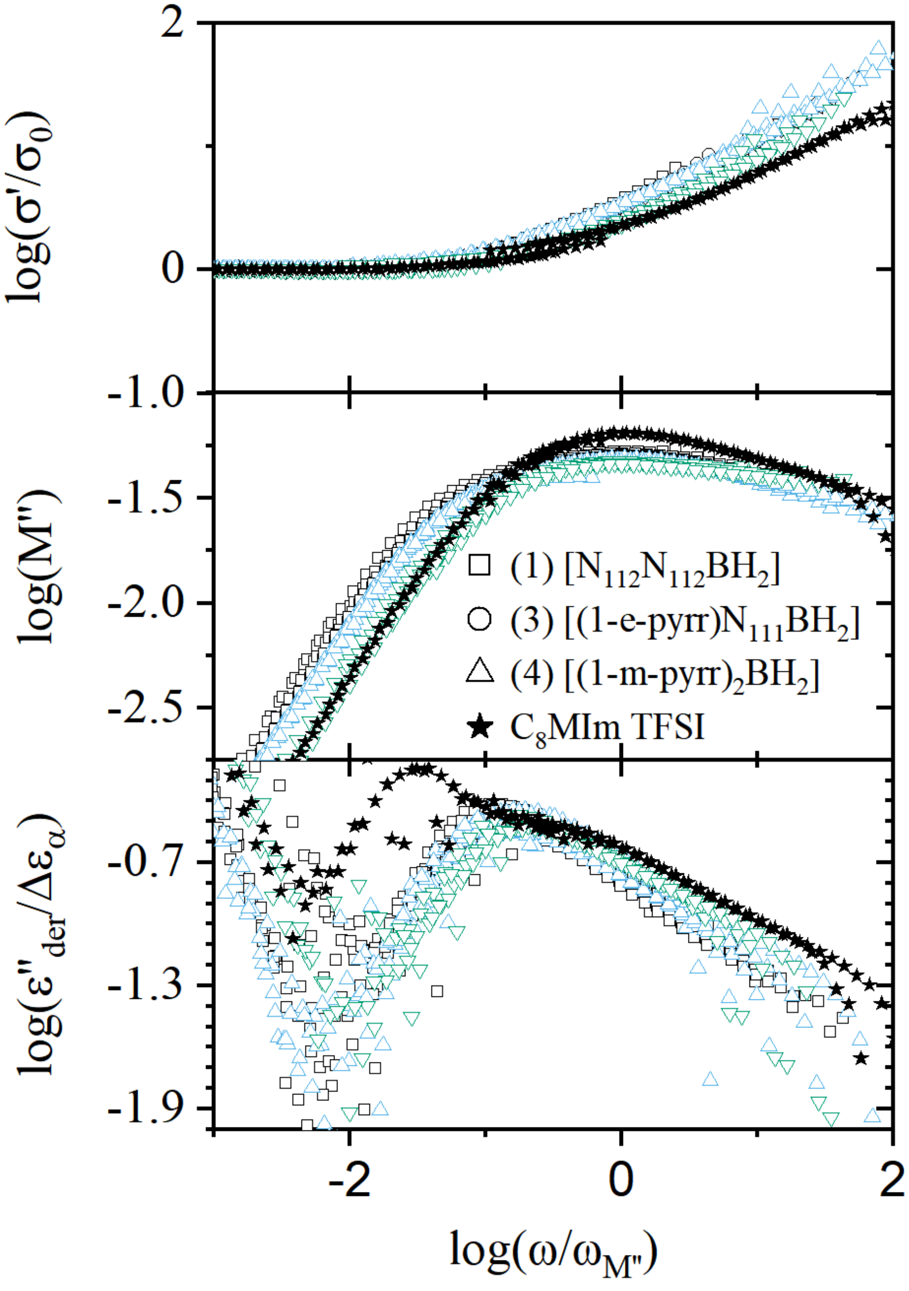

Figure S12. Broadband dielectric spectra of BIL **1**, BIL **3**, BIL **4**, and C_8MIm TFSI *versus* frequency normalized by the peak frequency of the imaginary part of complex electric modulus, $\omega_{M''}$.

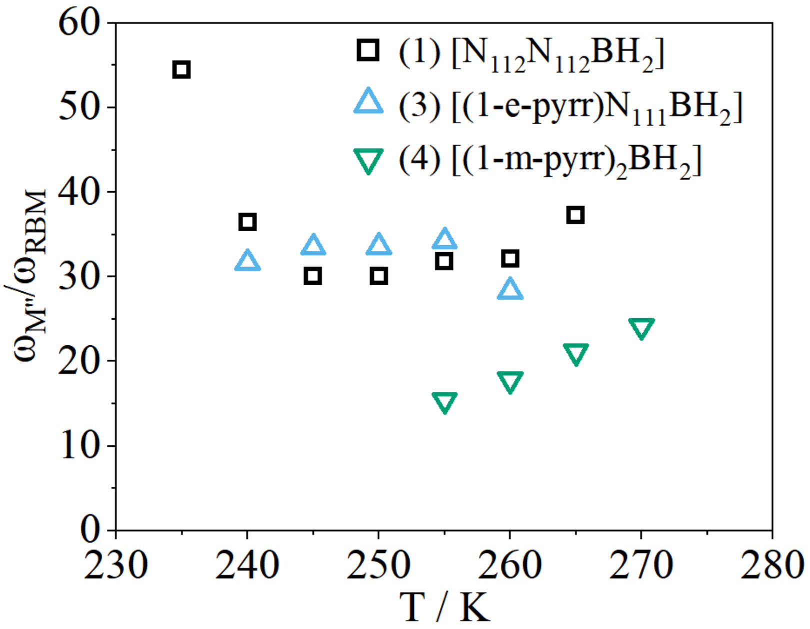

Figure S13. Broadband dielectric spectra of BIL **1**, BIL **3**, BIL **4**, and C_8MIm TFSI *versus* frequency normalized by the peak frequency of the imaginary part of complex electric modulus, $\omega_{M''}$.

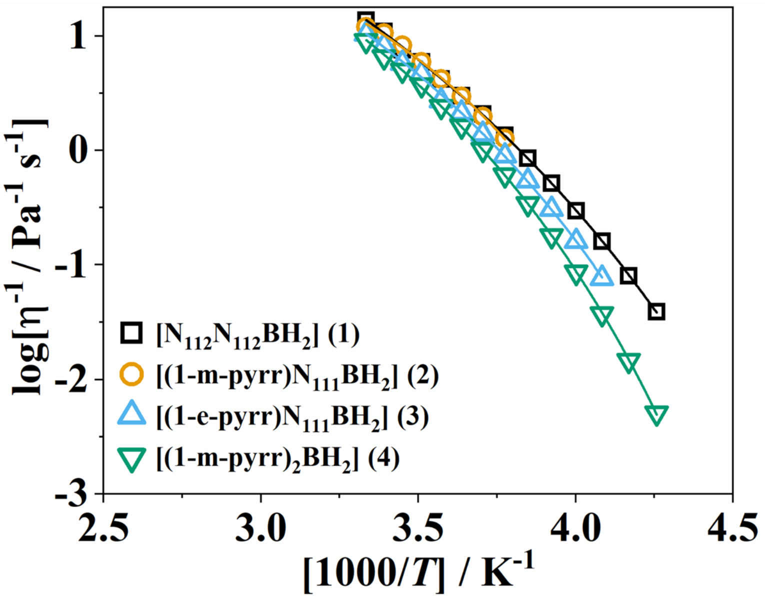

Figure S14. Temperature-dependent fluidities for BILs **1-4**. Fluidities were calculated from the temperature-dependent zero-shear viscosities measured on an Anton Parr rheometer equip with an environmental temperature controller. Solid lines are fits from the Vogel-Fulcher-Tammann equation (values from the fit are provided in Table S1).

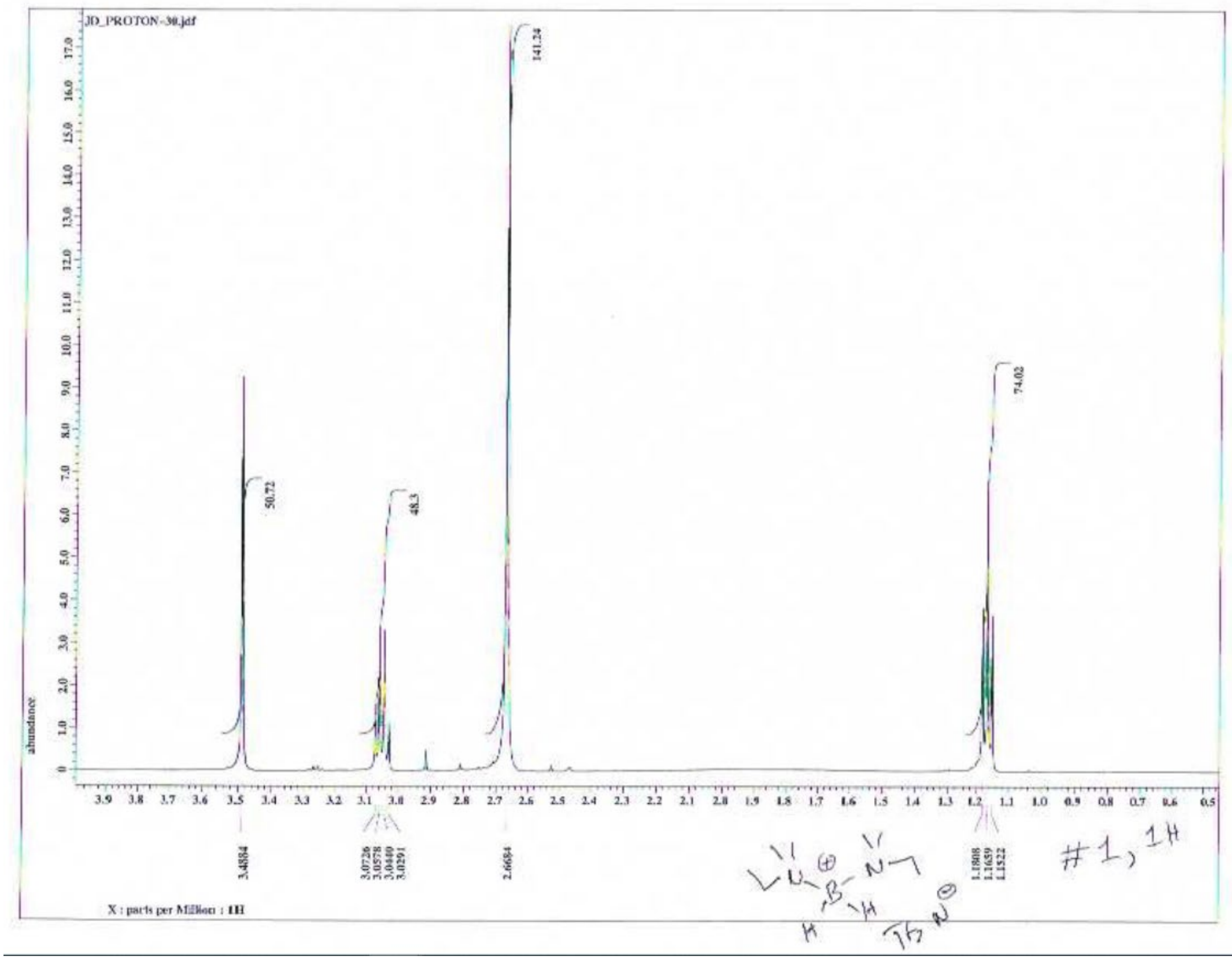

Figure S15. ^{1}H-NMR for BIL **1**.

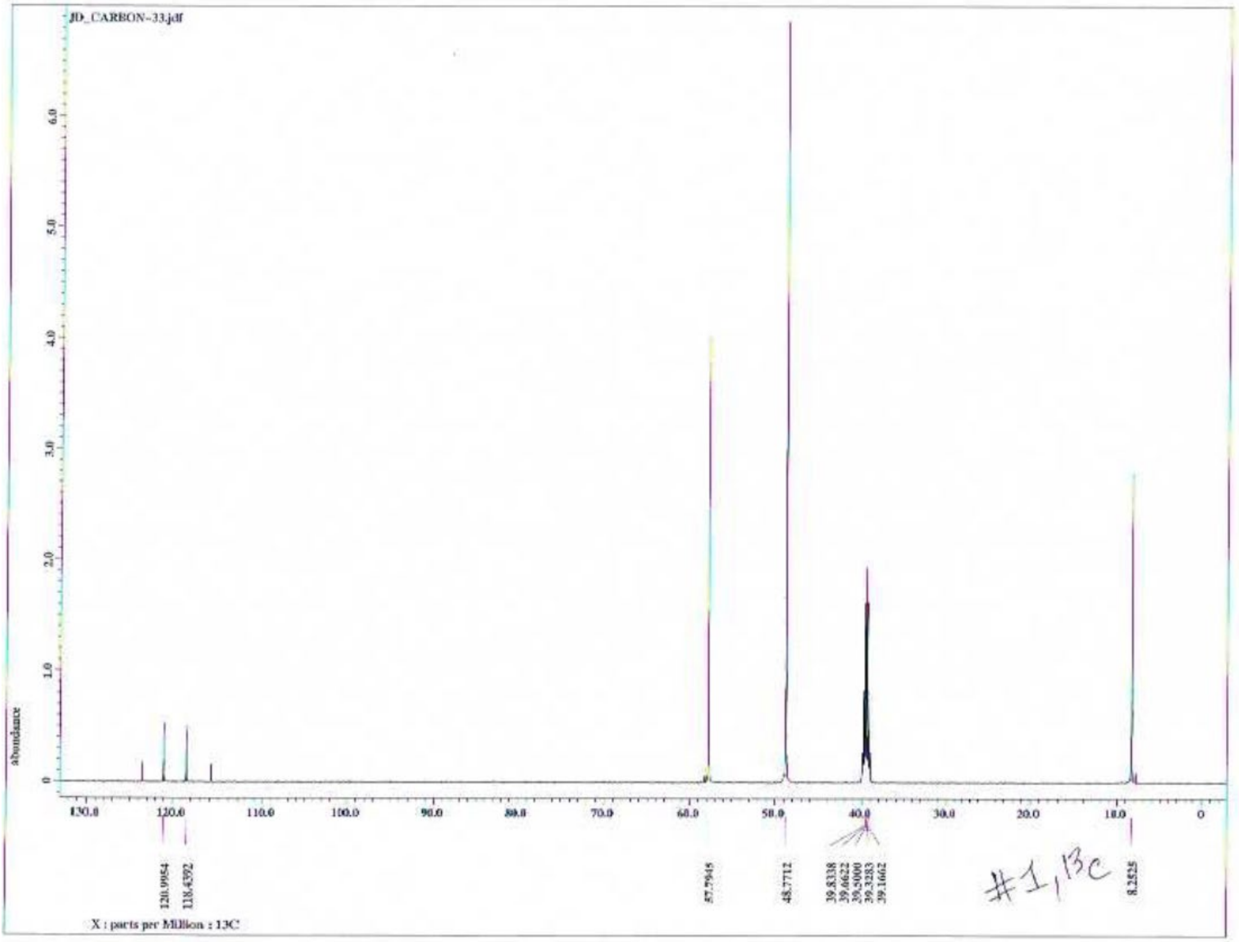

Figure S16. ^{13}C-NMR for BIL **1**.

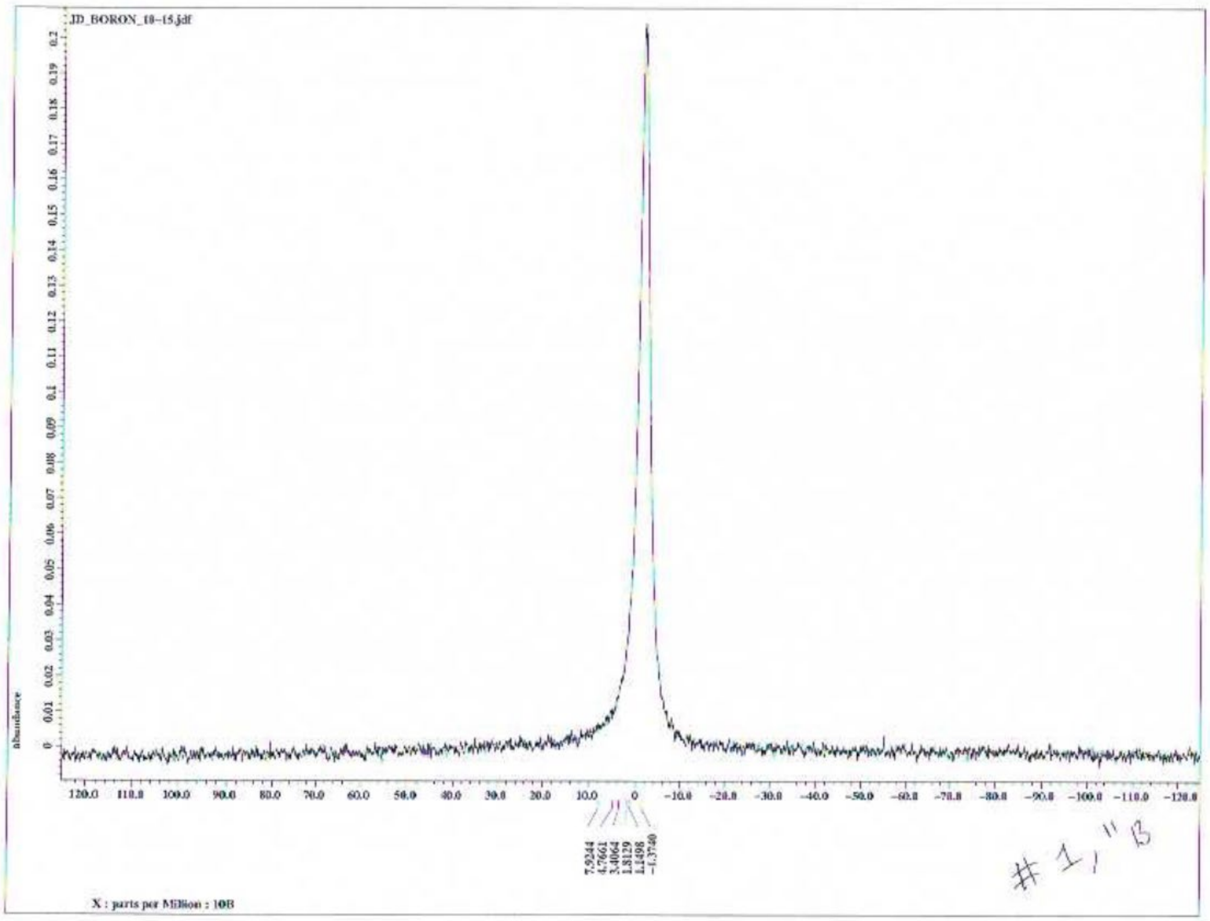

Figure S17. ^{11}B-NMR for BIL **1**.

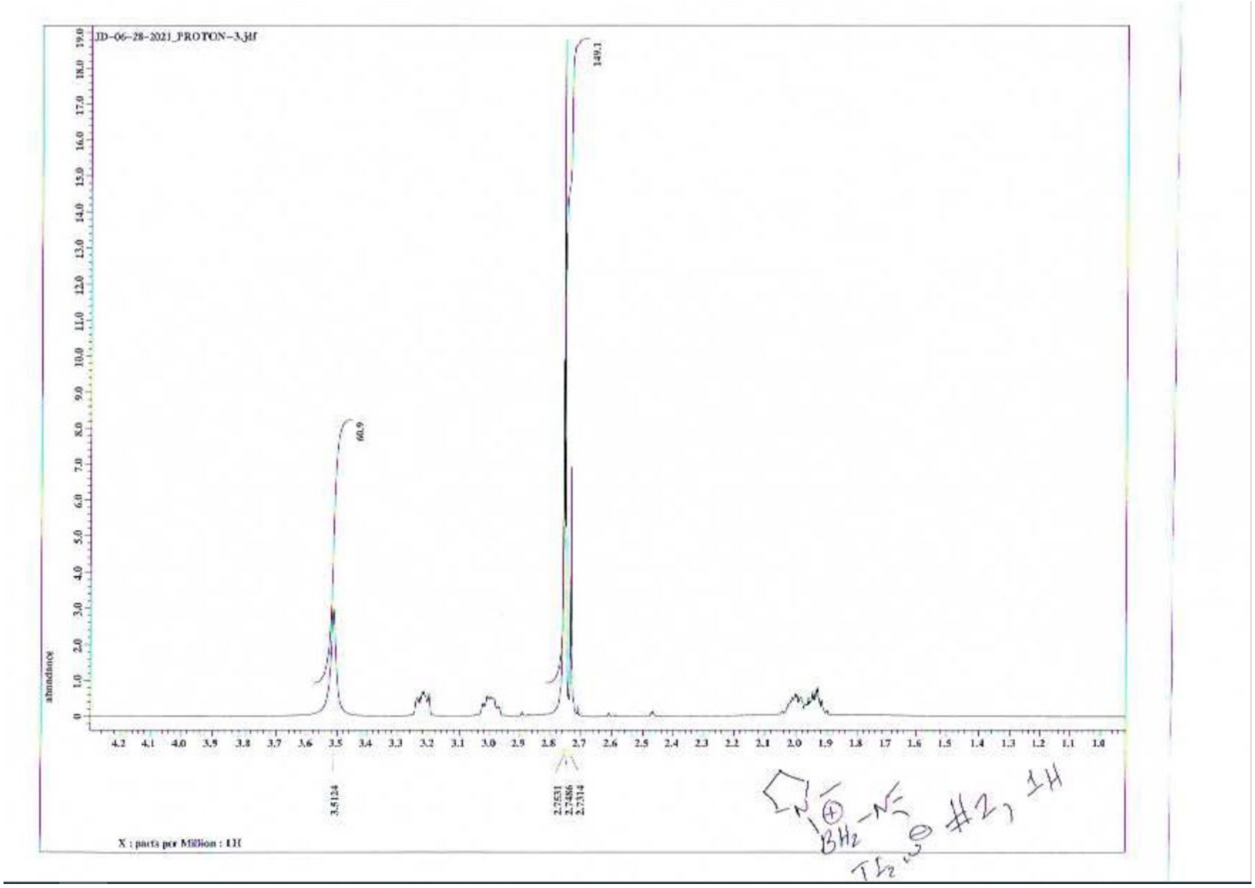

Figure S18. ^{1}H-NMR for BIL **2**.

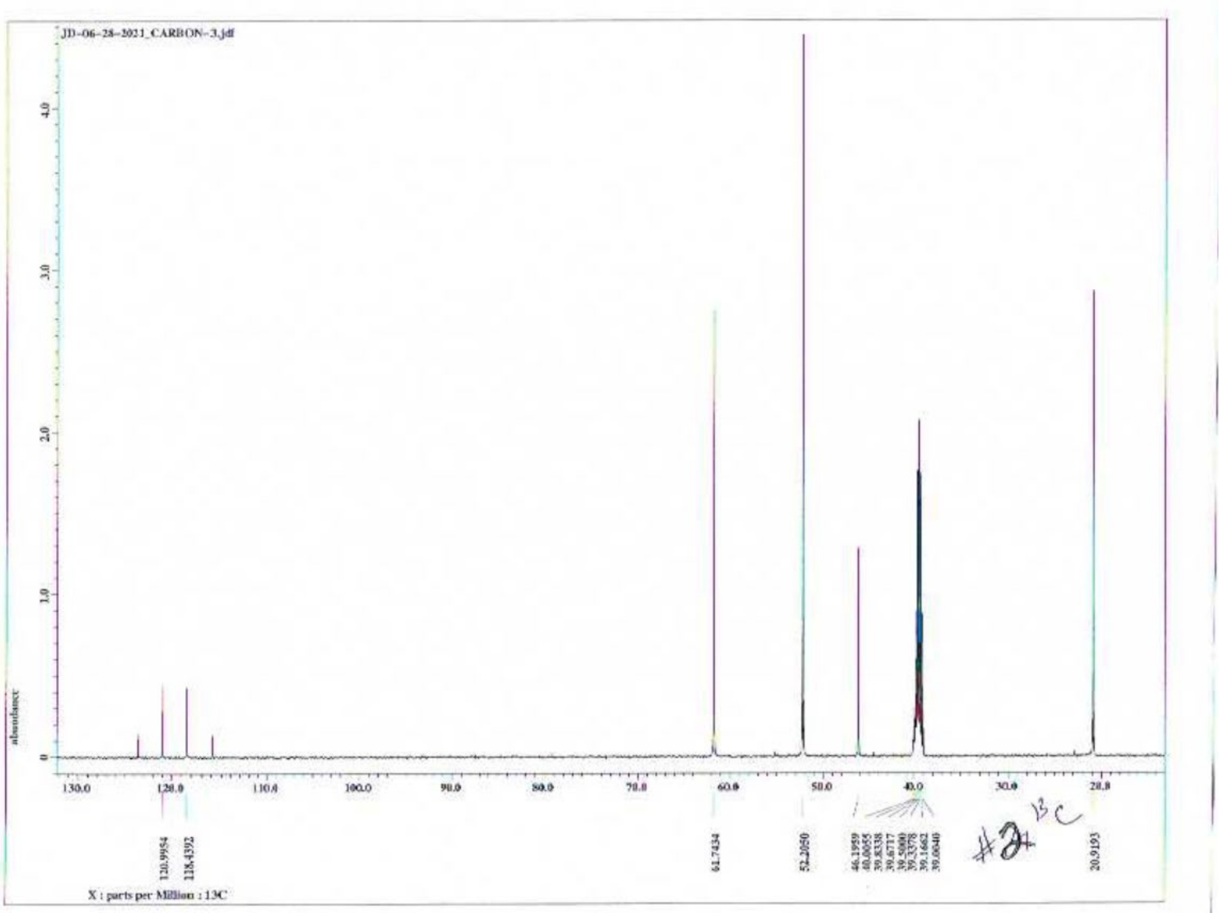

Figure S19. ^{13}C-NMR for BIL **2**.

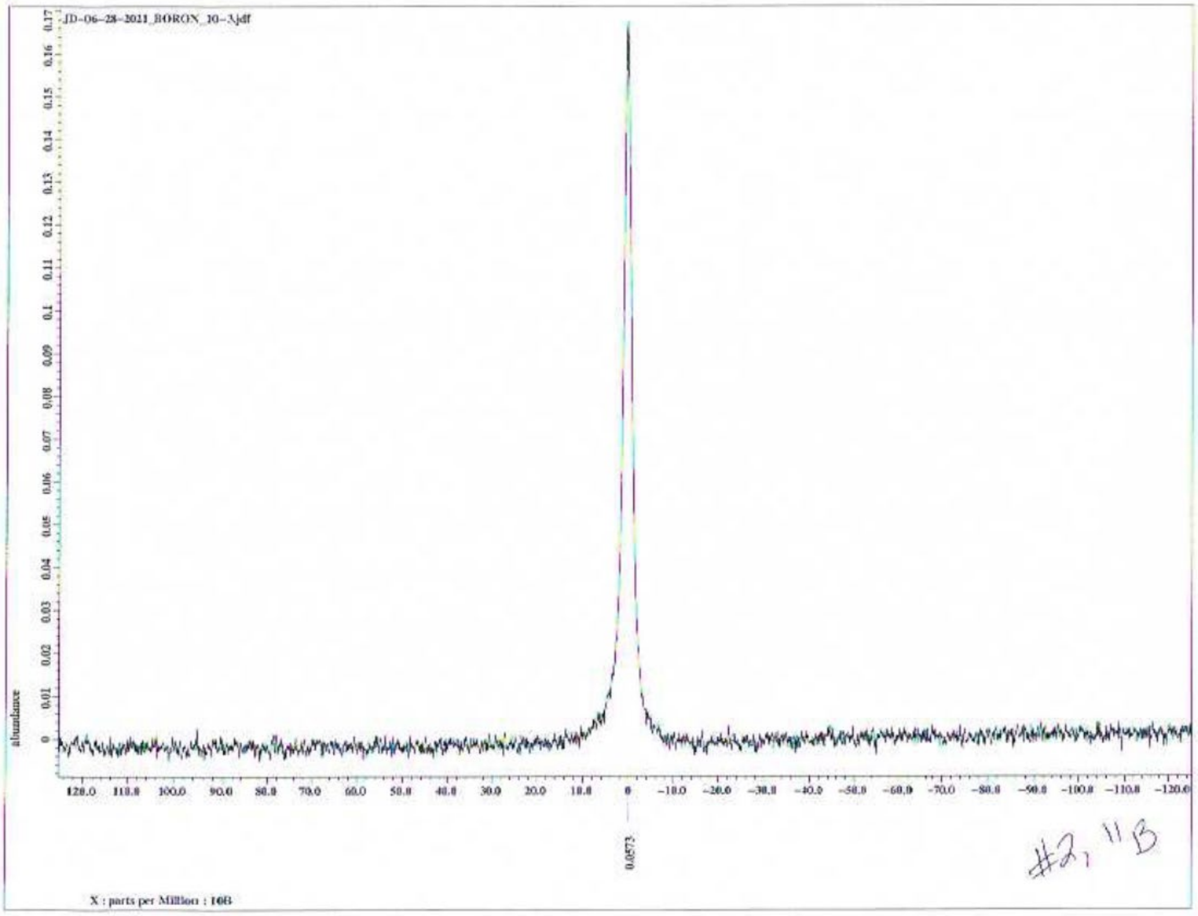

Figure S20. ^{11}B-NMR for BIL **2**.

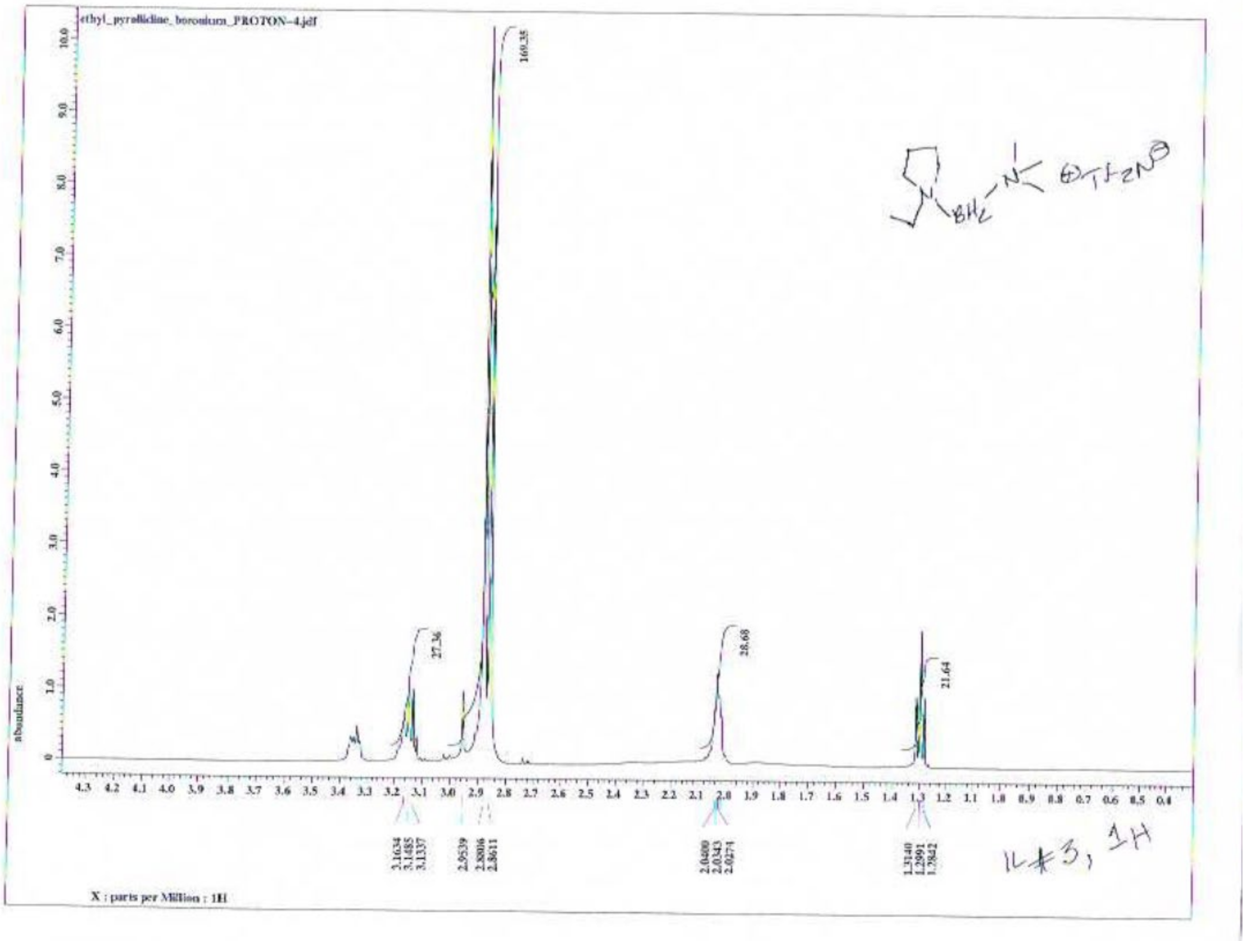

Figure S21. ^{1}H-NMR for BIL **3**.

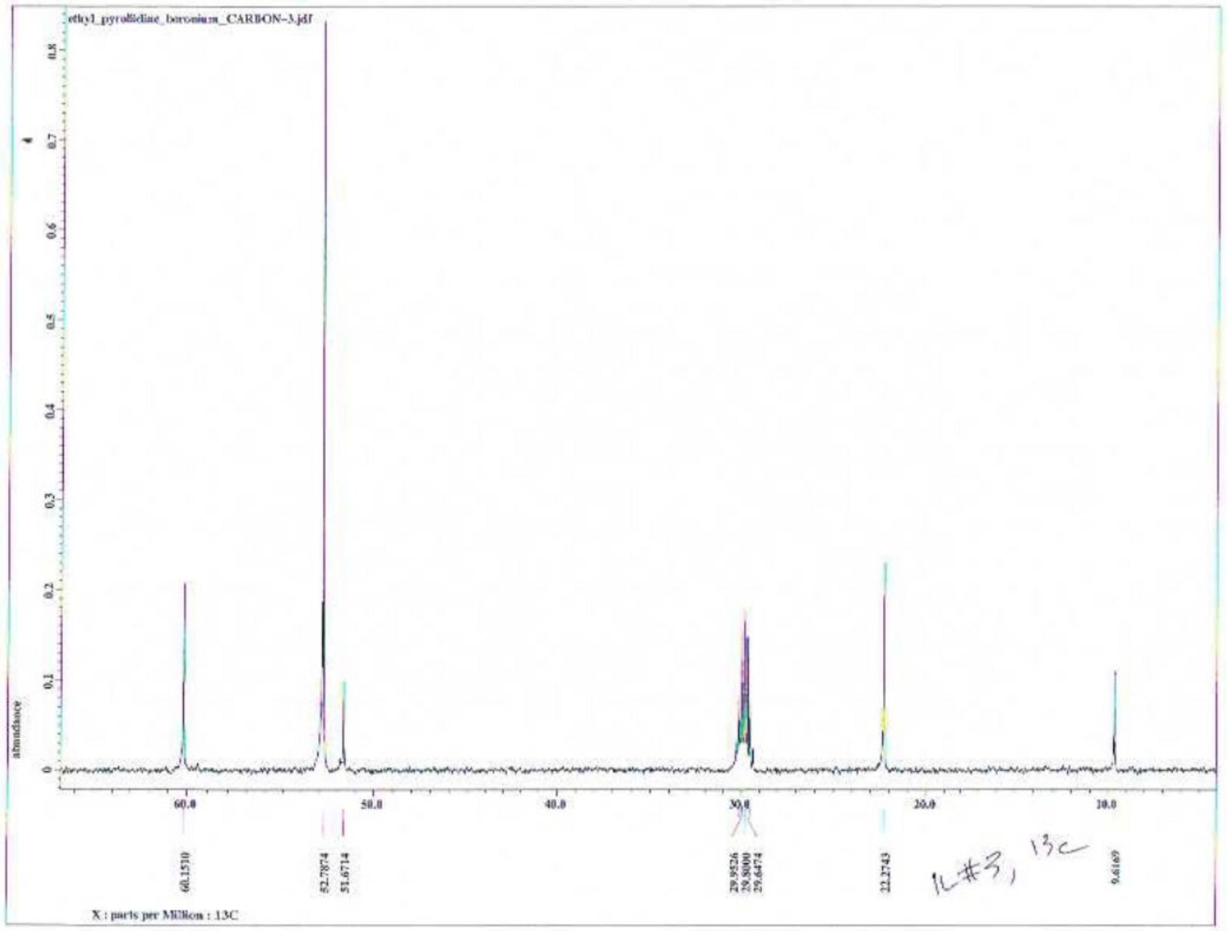

Figure S22. ^{13}C-NMR for BIL **3**.

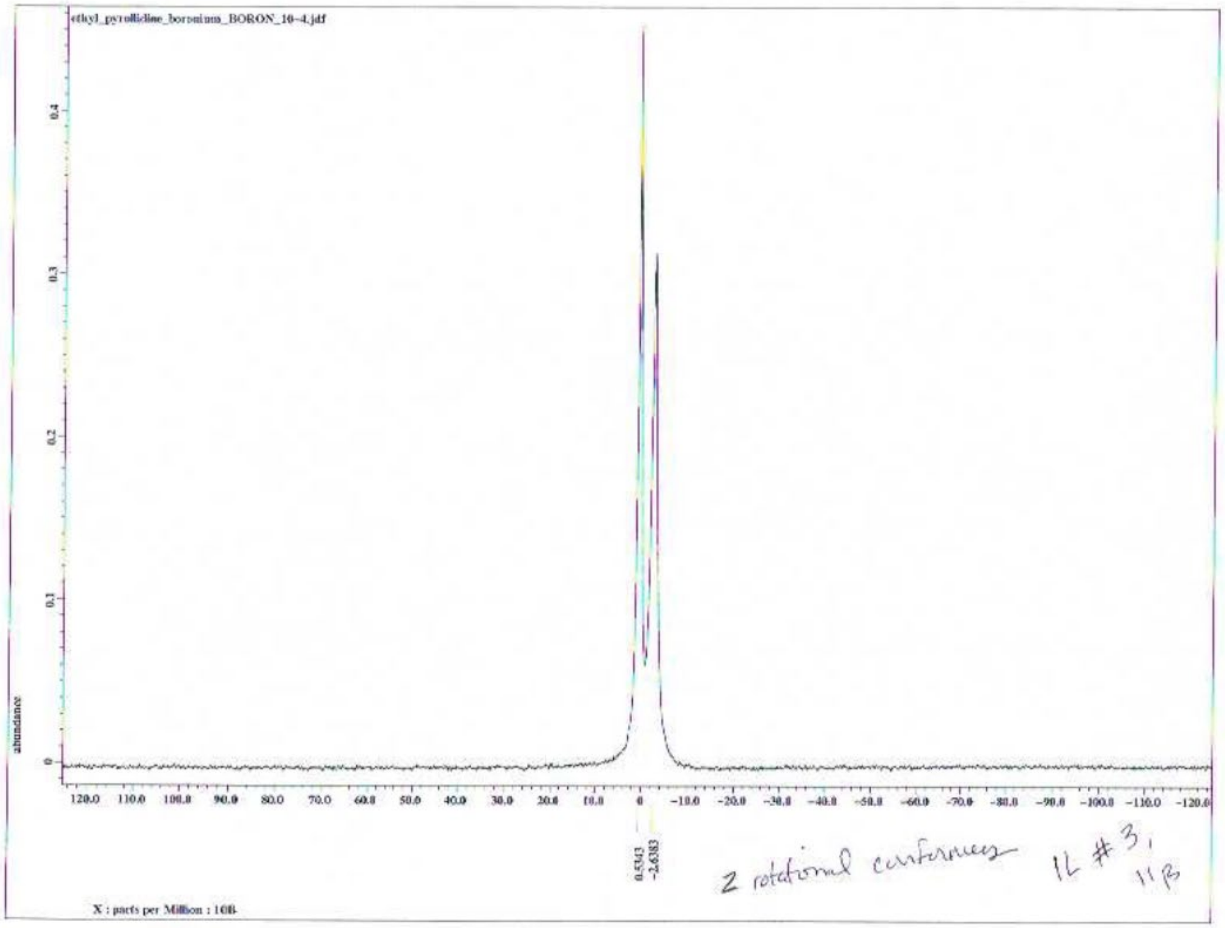

Figure S23. ^{11}B-NMR for BIL **3**.

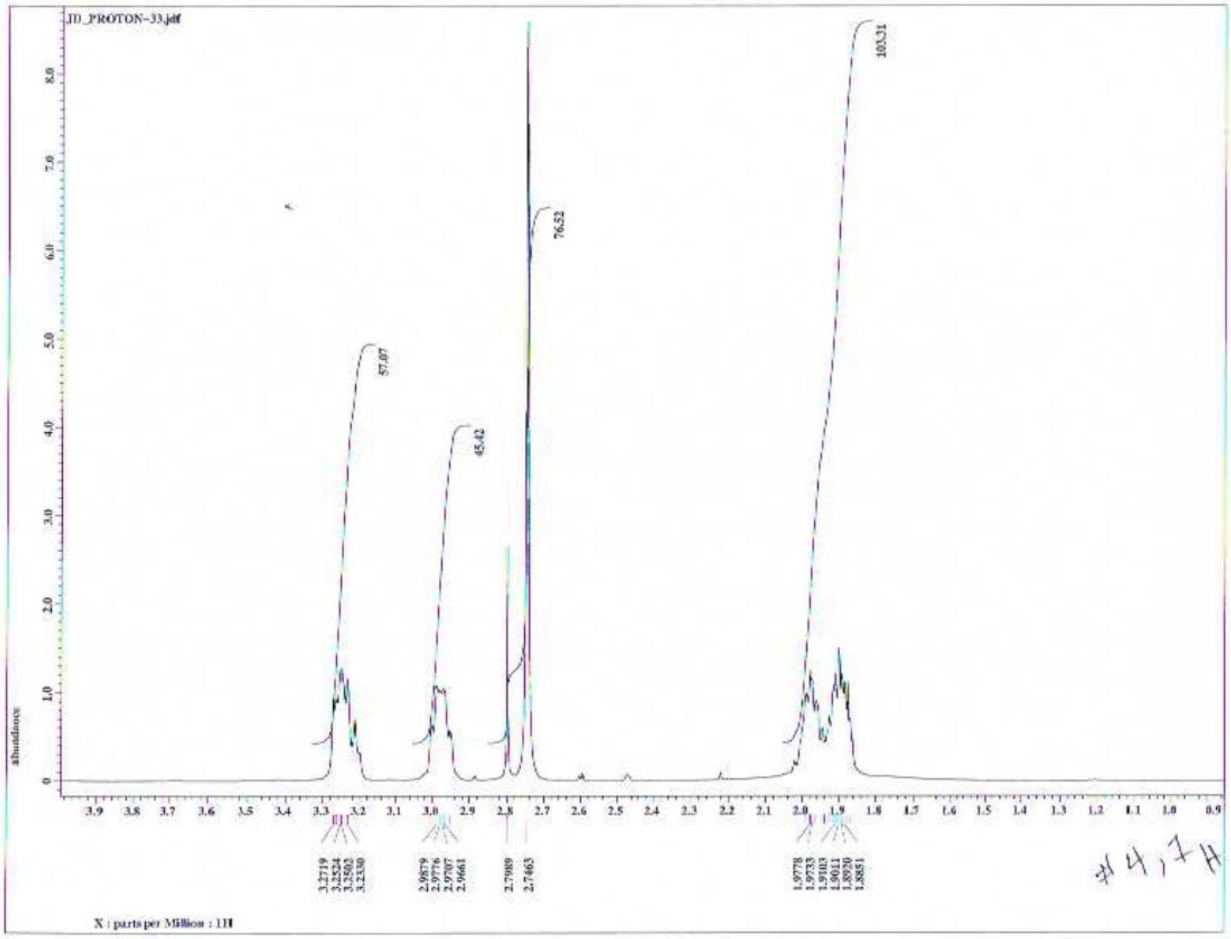

Figure S24. ^{1}H-NMR for BIL **4**.

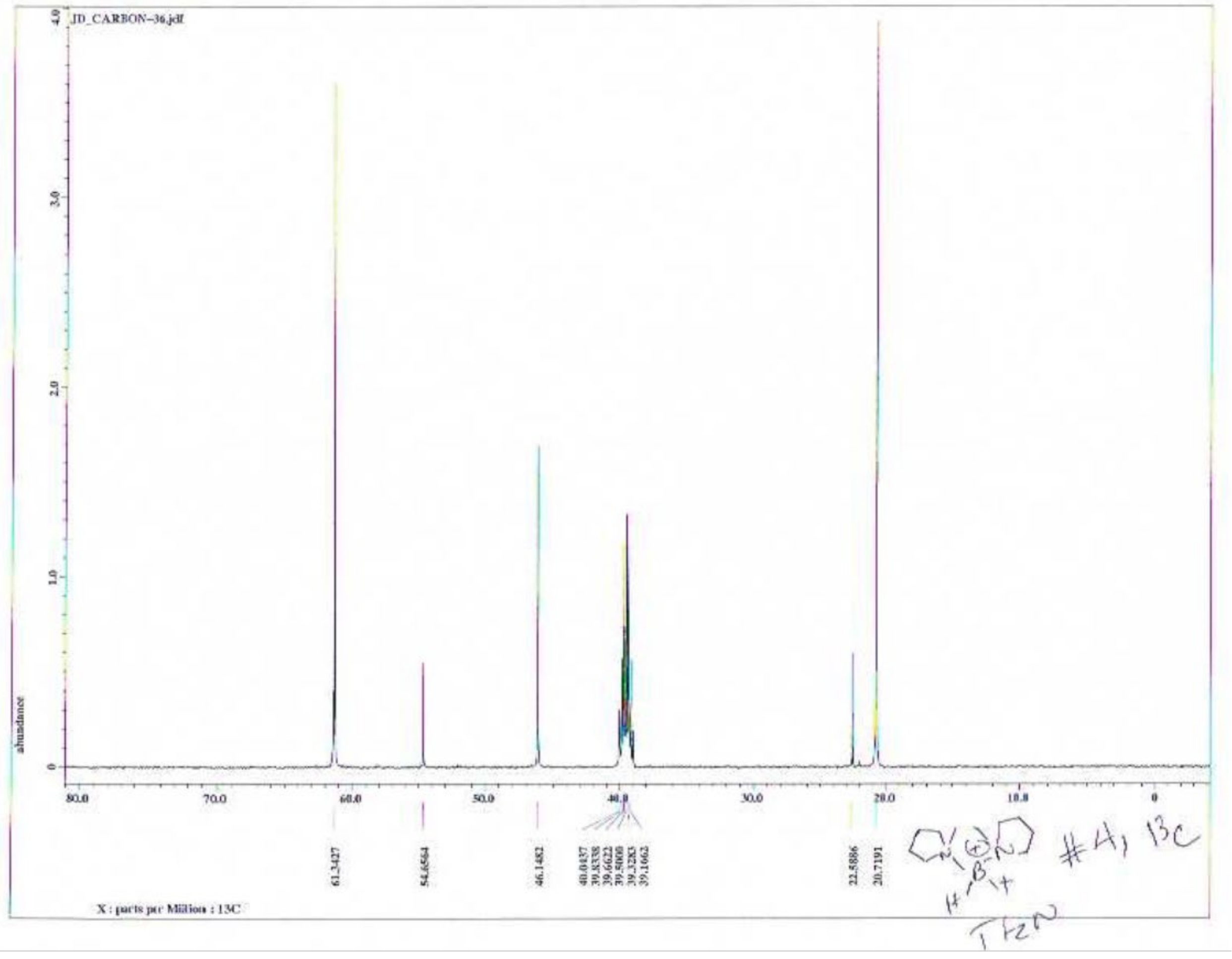

Figure S25. ^{13}C-NMR for BIL **4**.

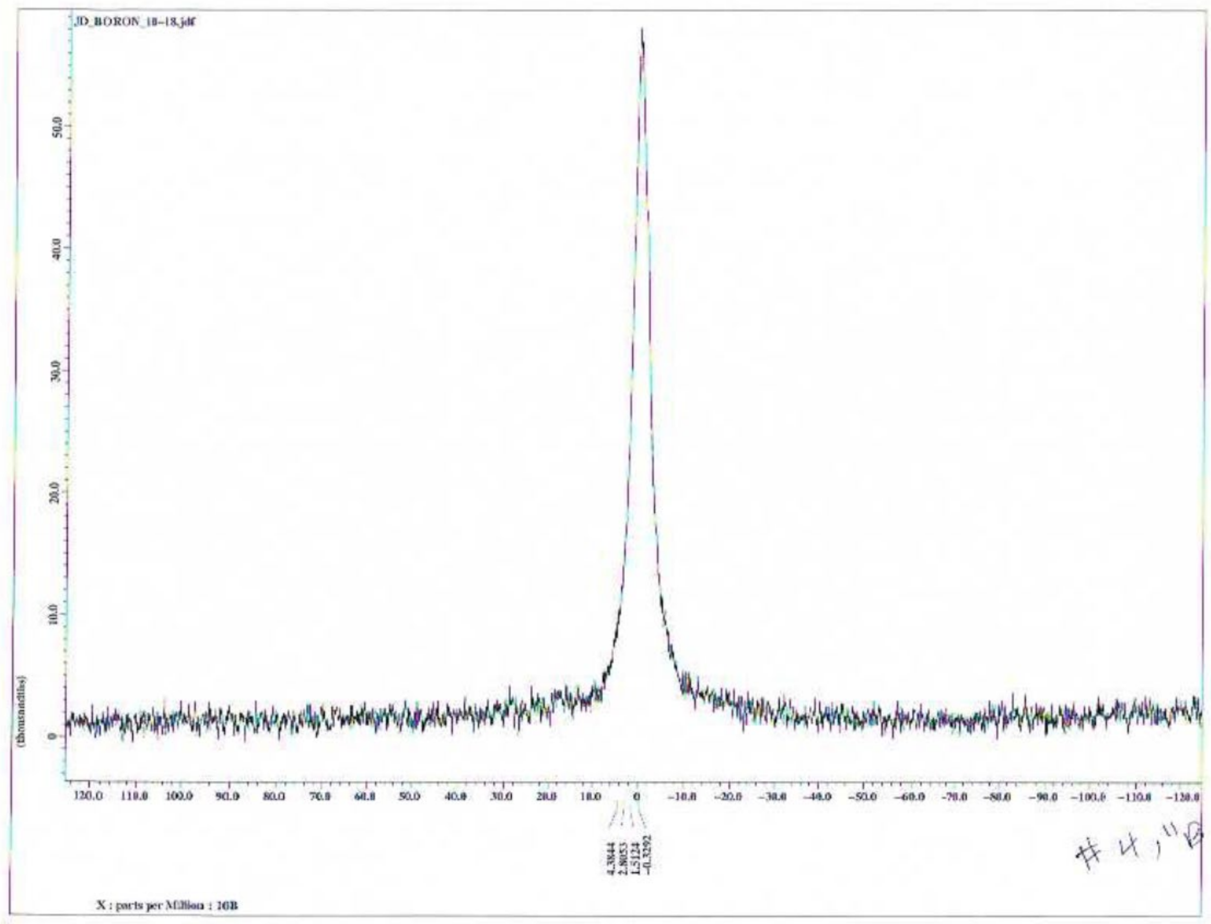

Figure S26. ^{11}B-NMR for BIL **4**.

[1] Bruker (2019). Apex3, Bruker AXS Inc.: Madison (WI), USA

[2] Bruker (2019). SAINT, Bruker AXS Inc.: Madison (WI), USA

[3] Bruker (2000-2003). SHELXTL suite of programs, Bruker AXS Inc.: Madison (WI), USA

[4] Sheldrick, G. A short history of SHELX. *Acta Crystallographica Section A.* **2008**, 64, 112-122

[5] Sheldrick, G. SHELXT - Integrated space-group and crystal-structure determination. *Acta Crystallographica Section A.* **2015**, 71, 3-8

[6] Sheldrick, G. Crystal structure refinement with SHELXL. *Acta Crystallographica Section C.* **2015**, 71, 3-8

[7] Hubschle, C. B., Sheldrick, G. M., Dittrich, B. ShelXle: a Qt graphical user interface for SHELXL. *J. Appl. Crystallogr.* **2011**, 44, 1281-1284

[8] Frisch, M. J., Trucks, G. W., Schlegel, H. B., Scuseria, G. E., Robb, M. A., Cheeseman, J. R., Scalmani, G., Barone, V., Petersson, G. A., Nakatsuji, H., Li, X., Caricato, M., Marenich, A. V., Bloino, J., Janesko, B. G., Gomperts, R., Mennucci, B., Hratchian, H. P., Ortiz, J. V., Izmaylov, A. F., Sonnenberg, J. L., Williams, Ding, F., Lipparini, F., Egidi,

F., Goings, J., Peng, B., Petrone, A., Henderson, T., Ranasinghe, D., Zakrzewski, V. G., Gao, J., Rega, N., Zheng, G., Liang, W., Hada, M., Ehara, M., Toyota, K., Fukuda, R., Hasegawa, J., Ishida, M., Nakajima, T., Honda, Y., Kitao, O., Nakai, H., Vreven, T., Throssell, K., Montgomery Jr., J. A., Peralta, J. E., Ogliaro, F., Bearpark, M. J., Heyd, J. J., Brothers, E. N., Kudin, K. N., Staroverov, V. N., Keith, T. A., Kobayashi, R., Normand, J., Raghavachari, K., Rendell, A. P., Burant, J. C., Iyengar, S. S., Tomasi, J., Cossi, M., Millam, J. M., Klene, M., Adamo, C., Cammi, R., Ochterski, J. W., Martin, R. L., Morokuma, K., Farkas, O., Foresman, J. B., Fox, D. J. (2016). Gaussian 16 Rev. C.01, Wallingford, CT

[9] Chai, J.-D., Head-Gordon, M. Long-range corrected hybrid density functionals with damped atom–atom dispersion corrections. *Phys. Chem. Chem. Phys.* **2008**, 10, 6615-6620

[10] Kendall, R. A., Dunning, T. H., Jr., Harrison, R. J. Electron affinities of the first-row atoms revisited. Systematic basis sets and wave functions. *J. Chem. Phys.* **1992**, 96, 6796-6806

[11] Baik, M. H., Friesner, R. A. Computing Redox Potentials in Solution: Density Functional Theory as A Tool for Rational Design of Redox Agents. *J. Phys. Chem. A.* **2002**, 106, 7407-7412

[12] Wavefunction, Inc. (2020). Spartan'20, Irvine, CA
